# In Situ UV–vis Quantification of Chlorobasicity in Binary Molten Chlorides

**DOI:** 10.1021/acs.jpcb.6c03131

**Published:** 2026-08-28

**Authors:** Kailee Buttice, Shubhojit Banerjee, Thiago J. Pinheiro dos Santos, Ellie Kim, Qiufeng Yang, Alejandro Ramos-Ballesteros, Sheng Dai, Ruchi Gakhar, Santanu Roy, Vyacheslav S. Bryantsev, Adrien Couet

**Affiliations:** † Department of Nuclear Engineering and Engineering Physics, 5228 University of Wisconsin-Madison, Madison, Wisconsin 53706, United States; ‡ Chemical Sciences Division, 6146 Oak Ridge National Laboratory, Oak Ridge, Tennessee 37830, United States; § Department of Chemistry, 312344 University of Tennessee, Knoxville, Tennessee 37916, United States; ∥ Advanced Technology of Molten Salts, 17212 Idaho National Laboratory, Idaho Falls, Idaho 83415, United States

## Abstract

Optical basicity measurements provide a basis for determining the relative chlorobasicity of a given molten salt composition. This study uses Bi^3+^ as a spectroscopic probe to investigate optical basicity across three binary molten chloride systems (LiCl-KCl, MgCl_2_–KCl, and ZnCl_2_–KCl) through in situ UV–vis spectroscopy measurements of the ^1^S_0_→^3^P_1_ transition. LiCl-KCl systems exhibit the highest optical basicity with minimal compositional dependence, while both divalent systems show stronger, yet nonlinear, dependence that is reminiscent of acid–base titration curves. The influence of cation electronegativity and polarizing power (Li^+^ < Mg^2+^ < Zn^2+^) directly reflects the basicity curves, with ZnCl_2_–KCl displaying the steepest gradients and lowest basicity values. Nonlinear dependencies in divalent systems indicate formation of chloride-bridged network structures with coordination states dictated by the amount of KCl in a salt composition, which was further investigated and validated by molecular dynamics simulations of polymeric structures formed in ZnCl_2_ and MgCl_2_-based salts using machine learning force fields. The temperature dependence of chlorobasicity in terms of optical basicity was investigated, and a loose, positive correlation between the two was revealed. These findings establish Bi^3+^ as a sensitive probe for understanding electronic environments in molten chloride systems and provide insights for predicting the chemical behavior of molten salts for use in high-temperature environments.

## Graphical abstract

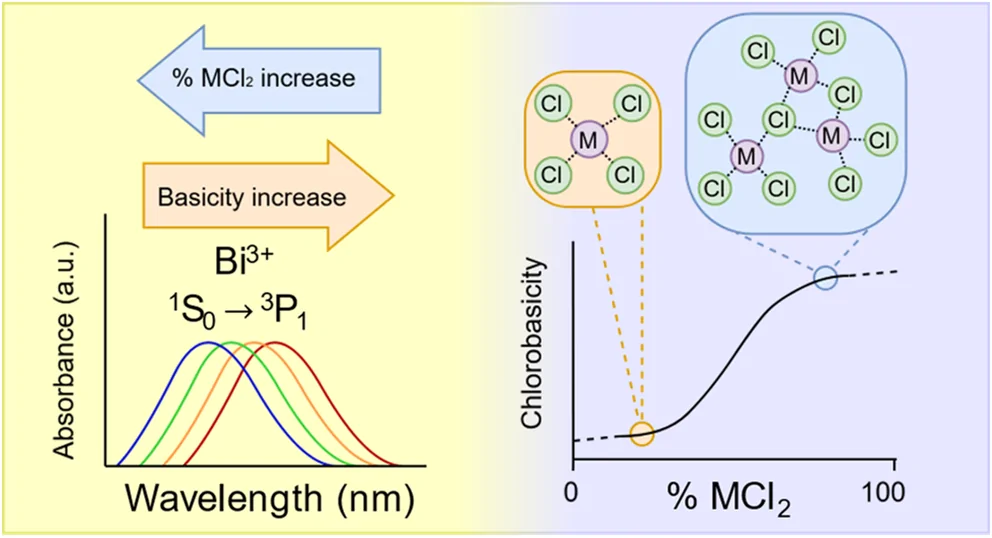

## Introduction

Understanding the fundamental acid–base chemistry of molten chloride salts is essential for predicting their behavior in the high-temperature energy applications, including molten salt reactors, concentrated solar power systems, and thermal energy storage.^1–6^ The chloride activity of these systems directly influences critical processes such as corrosion of structural materials, redox equilibria of dissolved species, and solvation behavior of metal ions.^7–12^ The relationship between chloride activity and chlorobasicity (or inversely, chloroacidity) is reflective of the Lewis acid–base type behavior in molten chloride systems. Chlorobasicity is a thermodynamic measure of chloride availability in a molten chloride melt, commonly quantified from complexation/speciation equilibria involving metal ions.^13,14^ Thus, high chlorobasicity corresponds to high chloride activity, *a*_Cl^–^_, an essential quantity for redox potential calculations in molten salt systems, which remains difficult to directly measure. Indeed, corrosion is influenced by chloride chemical potentials and chlorobasicity, which sets the thermodynamic driving force for the half-cell reactions below, and, in turn, the extent of corrosion. This is represented by half-cell reactions 1 and 2, resulting in overall reaction 3:^15^

R1 $$xM+yCl^{-}↔M_{x}Cl_{y}+ye^{-}$$

R2 $$\frac{y}{2}Cl_{2}+ye^{-}↔yCl^{-}$$

R3 $$xM+\frac{y}{2}Cl_{2}↔M_{x}Cl_{y}$$

Optical basicity (OB), derived from spectral shifts of dilute 5*d* ^10^6*s* ^2^ probe ions like Bi^3+^, Pb^2+^, and Tl^+^, provides a complementary spectroscopic measure of the chloride environment by describing the electron-donating capacity of the salt, i.e., the availability of electron density on Cl^–^ ions for interaction with other species within the melt. In terms of oxobasicity, the analogue to chlorobasicity in molten oxide glasses, salts, and slags for which OB measurements were first employed, more basic environments of high OB tend to promote oxide formation and passivation, while acidic melts of lower OB favor the dissolution of certain metal oxides.^3,16,17^ By analogy, in chloride molten salts, the chloride electron-donor environment quantified by OB is expected to influence the stability and speciation of dissolved metals. Such links are not yet fully established experimentally for chloride systems—a companion study to this work suggests a chlorobasicity–corrosion link for the Eu­(III)/Eu­(II) buffer and Ni-20Cr corrosion in LiCl-KCl vs MgCl_2_–KCl.^18^ However, connecting OB directly to corrosion rates, redox potentials, or metal speciation is left for future work. If this analogy holds, OB should be a useful parameter for understanding and controlling melt chemistry and corrosivity.

OB theory, pioneered by Duffy and Ingram in the 1970s, defines basicity as the average polarization of a probe ion’s surroundings, essentially quantifying the charge density donated to a probe by surrounding anions via the change in energy of the ^1^S_0_→^3^P_1_ electronic transition. The energy decrease, i.e., peak frequency decrease, is caused by the nephelauxetic effect, which describes the extent of a probe ion’s orbital expansion and resulting decrease in interelectronic repulsion.^19–21^ This technique has been particularly valuable for characterizing oxide glass and slag systems, where empirical correlations have been established between OB values and properties such as viscosity,^22,23^ ionic conductivity,^24,25^ and metal solubility.^26,27^ The OB of a salt may be calculated using eq 4, in which the frequencies ν_free_, ν_exp_, and ν_ref_ represent those of the “free” gaseous Bi^3+^ ion,^28,29^ the experimental Bi^3+^ transition in a sample salt, and the Bi^3+^ transition in an ultimately basic reference melt, which is LiCl-KCl eutectic in this work. The resulting OB (mathematically referred to as Λ) value lies between 0 and 1, as the “free” Bi^3+^ exists in a theoretical environment of zero electronic donation, and the reference salt is chosen for its maximum ionicity.

1 $$\Lambda =\frac{\nu_{free}-\nu_{\exp}}{\nu_{free}-\nu_{ref}}=\frac{56,000\;cm^{-1}-\nu_{\exp}}{56,000\;cm^{-1}-\nu_{ref}}$$

Throughout this work, we use *chlorobasicity* to refer to the underlying chemical property describing chloride electron-donating capacity and *optical basicity* to refer specifically to the spectroscopic quantity derived from the Bi^3+^ transition energy that serves as an experimental proxy for it. Recently, chlorobasicity has also been measured via electrochemically determined chloride activity.^30^

Extensive application of OB to molten chloride systems has only recently been done. Sharpless et al. established OB as a spectroscopically accessible measure of Lewis basicity for molten chloride salt mixtures and examined its temperature dependence, while a related review by the same group surveyed the broader OB literature, highlighting its potential relevance to molten salt corrosion.^17,31^ These studies demonstrated the feasibility of OB application to a suite of chloride salts, as well as its dependence on cation identity and temperature, providing modern benchmark values for OB in chloride systems. The present work builds on the foundation set by Duffy et al. and Sharpless et al., both experimentally and computationally. Here, Bi^3+^ is employed as a probe ion, chosen for its lower and more accessible ^1^S_0_→^3^P_1_ transition energy relative to Pb^2+^ and Tl^+^, to establish comprehensive OB scales for three binary chloride systems spanning monovalent (LiCl-KCl) and divalent (MgCl_2_–KCl, ZnCl_2_–KCl) compositions. This systematic approach incorporates molecular dynamics (MD) simulations using machine learning force fields (MLFF) and polarizable ion models (PIM) to reveal composition-dependent melt structure, coordination preference, speciation, and network-forming ability, which has not previously been included in an OB study. This work aims to substantiate OB as a robust descriptor linking composition and temperature to melt structure and chloride electron-donor strength. Corrosion, redox equilibria, and dissolved metal speciation are each expected to depend on that same electron-donor environment. Consequentially, a validated OB scale could eventually assist in interpreting redox behavior across different salts and inform the design of corrosion-resistant melts, though the exact nature of those connections remains to be established in future work.

## Experimental Methods

### Salt Preparation

The MgCl_2_–KCl salts were purified and provided by the University of Tennessee—Knoxville. Magnesium chloride (Alfa Aesar, 99% purity) was further purified by sparging with dry air at 800 °C for 2 h, followed by fractional distillation at 900 °C for 24 h, and a final sparging with chlorine gas at 800 °C for 1 h. Potassium chloride (Sigma-Aldrich, 99.999% purity) was dehydrated at 500 °C for 2 h. Further details on the chloride salt purification process and its efficacy can be found in prior work.^32^ The purified MgCl_2_ and KCl were mixed in molar ratios of *X*:100–*X* (*X* = 30, 40, 50, 60, 65, 70) in a fused silica tube fitted with a #15 Ace-Thred fitting. The mixture was heated to 800 °C under vacuum until it appeared homogeneous. Salts were sealed in an Ar glovebox in airtight jars containing desiccant and shipped to the University of Wisconsin–Madison (UW-M).

The zinc and lithium salts are less hygroscopic than magnesium salts,^33^ so high-purity anhydrous commercial salts were used directly. ZnCl_2_–KCl salts were prepared by mixing powdered ZnCl_2_ (Thermo Scientific, 99.95% purity) and KCl (Thermo Scientific, 99.999% purity) in molar ratios of *X*:100–*X* (*X* = 30, 40, 47.5, 54.7, 60, 65, 70, 75, 80). The LiCl-KCl salts were prepared by mixing powdered LiCl (Sigma-Aldrich, ≥ 99% purity) and KCl powders in molar ratios of *X*:100–*X* (*X* = 50, 60, 70, 80). The commercial salts were baked for 12 h at 200 °C to ensure dehydration prior to use but were not fused prior to melting in the cuvettes for measurement.

Optical basicity is a function of the Bi^3+^ peak wavelength rather than its intensity; thus, the concentration of the probe does not necessarily need to be extremely precise. To confirm this, a sample of ∼1 mM BiCl_3_ dissolved in eutectic LiCl-KCl was heated at 550 °C for an extended measurement duration, recording a spectrum every minute for 140 min to assess the depletion of Bi^3+^ over time and its effect on peak position (Figures S9–S11). The peak wavelength did not vary >1 nm over the duration of these measurements, while the peak amplitude decreased by ∼70%. Still, the high molar absorptivity of bismuth in the UV regime requires a very low concentration of the probe ion, so serial dilution is required when preparing small electrolyte volumes. For the initial dilution, BiCl_3_ (Sigma-Aldrich, 99.999% purity) was added to KCl at 800 °C in a covered glassy carbon crucible, to a concentration of 400 mM. The BiCl_3_ was added outside the furnace, immediately stirred into the molten KCl by swirling the crucible, and the salt was allowed to cool in the covered crucible at room temperature prior to removal. Due to the high volatility of BiCl_3_ and despite fusing in a closed crucible, it is likely that the concentration fell below 400 mM, but it is not known exactly. Each MgCl_2_, LiCl, or ZnCl_2_ -KCl salt composition was doped with the diluted BiCl_3_ to a concentration of roughly 1 mM (∼0.01–0.02 wt %).

### Optical Basicity Measurements

UV–vis spectra of Bi^3+^ in molten salts were collected using a Cary 4000 spectrophotometer that was adapted for in situ measurements by coupling it to a high-temperature fiber optics assembly (custom-built from Thorlabs), which interfaced with an Ar atmosphere glovebox (<0.5 ppm of O_2_ and <0.1 ppm of H_2_O). A schematic of the experimental setup is shown in Figure 1. In quartz cuvettes with a path length of 1 cm, ∼0.001 g of the diluted BiCl_3_–KCl was added to ∼3 g of base salt (LiCl-KCl, ZnCl_2_–KCl, MgCl_2_–KCl). The cuvettes were topped with fitted boron nitride lids to minimize salt volatilization, and the salt was heated until melted (550 °C for LiCl-KCl and MgCl_2_–KCl salts, 450 °C for ZnCl_2_–KCl salts) in a custom spectroscopy furnace (Figure 1). After allowing the salt to equilibrate at each measured temperature until the spectrum reached a static shape, ∼5 min, UV–vis spectra were collected for each sample with a resolution of 1 nm and a scan rate of 10 nm/s. Spectra for the Mg^2+^ salts were measured at 500, 525, 550, 575, and 585 °C. Spectra for the Li^+^ salts were measured at 450, 475, 500, 525, and 550 °C, while spectra for the Zn^2+^ salts were measured at 350, 400, and 450 °C due to the volatility of ZnCl_2_-containing salts at higher temperatures.^34^ Data processing was completed using Origin 2018. The spectrum for each salt composition without bismuth was subtracted from the Bi^3+^-doped spectrum at each temperature to isolate the Bi^3+^ peak, which was then fitted to Origin’s bi-Gaussian function to determine peak position and associated error. The bi-Gaussian fit was selected due to the asymmetry of the peaks induced by overlap with the ^1^S_0_→^1^P_1_ transition (in the deep UV), reduced spectral efficiency, and Rayleigh scattering as the optics probed deeper into the UV regime.

**Figure 1:**
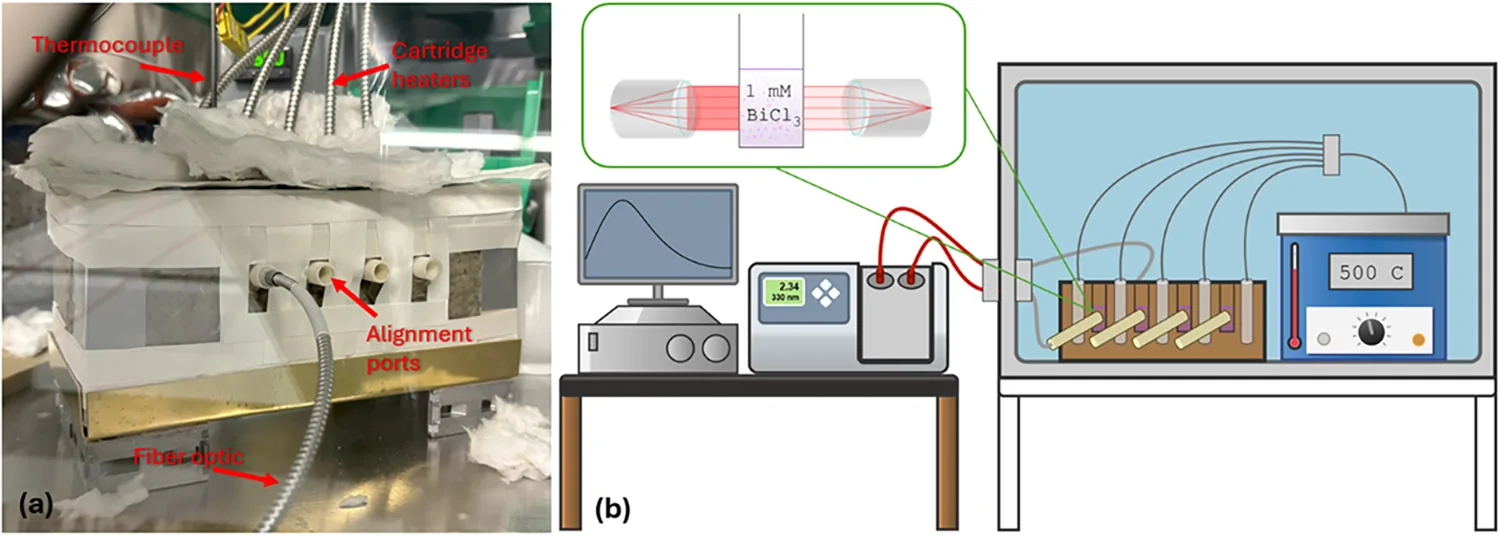
(a) In situ UV–vis spectroscopy furnace. Fiber optics is aligned at the beam inlet and outlet positions using alumina crucibles attached to a brass heat transfer block used to contain the cuvettes. The unit is heated using cartridge heaters powered by a PID controller within the glovebox. (b) Schematic of the UV–vis spectroscopy experimental setup. The spectrophotometer sits outside of the glovebox and is interfaced with the heater and samples inside the glovebox with fiber optic cables.

Despite experiments taking place in an inert Ar atmosphere, there is also the potential for Bi^3+^ to exist in the form of BiOCl in the presence of trace impurities from the commercial salt or leached from the sapphire cuvettes. BiOCl is soluble up to ∼12 wt % at 650 °C in molten CaCl_2_–NaCl-BaCl_2;_ ^35^ thus, we would not expect, nor did we observe, any precipitation of BiOCl in our salts, leaving bismuth to remain in ionic solution as Bi^3+^. The spectral behavior of Bi_2_O_3_ dissolved in aqueous HCl solutions does not dramatically differ from that which has been observed in molten chloride systems – the characteristic ^1^S_0_→^1^P_1_ peak still occurs at ∼320–330 nm, and it remains sensitive to the acid–base environment of its host solution.^36^ While we have not performed quantitative analysis of oxygen content in the salts measured here, there is not expected to be a significant disruption to measured OB caused by trace dissolved oxygen.

## Computational Methods

### Molecular Dynamics Simulations of ZnCl_2_–KCl Mixtures

Molecular dynamics (MD) simulations of ZnCl_2_–KCl mixtures at various compositions were performed using machine learning force fields (MLFFs) trained on density functional theory (DFT) calculations. Both MLFF and DFT-based simulations were carried out in the Vienna Ab initio Simulation Package (VASP).^37–39^ The MLFF training employed the gradient-corrected Perdew–Burke–Ernzerhof (PBE) exchange–correlation functional^40–42^ with dispersion interactions included via Grimme’s DFT-D3­(BJ) method.^43^ The interaction between valence electrons and atomic cores was described using the projector augmented-wave (PAW) method.^44,45^ GW-compatible PAW data sets were employed for all elements.^46^ In the PAW method, 20, 9, and 7 electrons were treated explicitly (including semicore states) for Zn (3s^2^ 3p^6^ 3d^10^ 4s^2^), K (3s^2^ 3p^6^ 4s^1^), and Cl (3s^2^ 3p^5^), respectively. All calculations employed a plane-wave basis set with a kinetic-energy cutoff of 600 eV. An electronic energy convergence threshold was set to 10^–5^ eV.

Training configurations were selected using VASP’s on-the-fly kernel-based machine learning scheme, which employs Bayesian linear regression to fit the model and estimate prediction uncertainties.^47,48^ In this approach, only structures with high predicted errors were added to the training set, substantially reducing the number of DFT calculations required. To better sample the Zn–Zn coordination space, we performed metadynamics in VASP using a continuous Zn–Zn coordination number as the collective variable *W*, which is given by eq 5,

2 $$W=\frac{1}{2}\sum_{i=1}^{N_{\mathrm{Zn}}}\sum_{j\neq i;\;j=1}^{N_{\mathrm{Zn}}}\frac{{1-\left(R_{\mathrm{ij}}/c\right)}^{M}}{1-{\left(R_{\mathrm{ij}}/c\right)}^{N}}$$

where *N*_Zn_ is the total number of Zn^2+^ ions in the simulation cell, *R*_*ij*_ is the distance between ions *i* and *j*, *c* is the cutoff distance, and *M* = 12 and *N* = 24 are the exponents that control the smoothness of the coordination function *W*. The cutoff distance for this coordination number was chosen as the first minimum of the Zn–Zn radial distribution function from unbiased MD, which allowed us to explore a broader range of Zn–Zn coordination motifs.

MLFFs were generated for compositions *X*_ZnCl_2__ = 0.33, 0.40, 0.45, 0.50, 0.55, and 0.67. Each production system was composed of roughly 250 atoms, and simulations were carried out at 450 °C. MLFF-MD simulations were performed using a 1 fs time step. Equilibrium volumes were determined in the isobaric–isothermal (NpT) ensemble using 1 ns of equilibration followed by 2 ns of production. The Parrinello–Rahman barostat^49,50^ maintained the pressure at 1 bar with a friction coefficient of 5 ps^–1^ and a fictitious lattice mass of 1000 amu, and temperatures were controlled by a Langevin thermostat^51^ with a friction coefficient of 1 ps^–1^. Table 1 summarizes the equilibrium densities of ZnCl_2_–KCl mixtures at different compositions at 450 °C. The results are in reasonable agreement with experimental values.^52^ The observed discrepancy reflects the usual shortcomings of DFT-based methods in capturing dispersion interactions.

**Table 1:** Densities of ZnCl_2_–KCl Mixtures at Different Compositions at 450 °C Obtained from the Trained MLFF, Compared with Experimental Data

*X*_ZnCl2_	ρ_MLFF_ (g/cm^3^)	ρ_Exp._ (g/cm^3^)^52^	Error (%)
0.333	2.07 ± 0.02	1.98	5
0.400	2.13 ± 0.02	2.04	4
0.450	2.17 ± 0.02	2.08	4
0.500	2.22 ± 0.02	2.11	5
0.552	2.28 ± 0.02	2.15	6
0.667	2.39 ± 0.02	2.25	6

Using these equilibrium densities (Table 1), initial configurations for each composition were prepared for simulations in the canonical (NVT) ensemble. These simulations included 500 ps of equilibration and 2.5 ns of production runs, with temperature controlled by the Nosé–Hoover thermostat^53^ using a fictitious Nosé mass of 2.8464 amu, corresponding to a temperature-oscillation frequency of approximately 100 fs. Atomic configurations were stored every 10 fs for data analysis.

### Molecular Dynamics Simulations of MgCl_2_–KCl Mixtures

MD simulations for the MgCl_2_–KCl mixture melts were performed at 0.33, 0.50, and 0.67 mole fractions of MgCl_2_ using the FIST module of the CP2K software.^54^ The initial structures for the simulations were generated by taking the snapshots from the ZnCl_2_–KCl mixtures trajectories and replacing Zn^2+^ with Mg^2+^. Using the polarizable ion model successfully deployed earlier to explain X-ray scattering features of these mixtures,^55–57^ we performed MD simulations in the NpT ensembles at 585 °C and 1 bar for 1 ns (ns) by employing the Nose–Hoover thermostat^53^ and barostat with a time constant of 1 ps. An MD time step of one femtosecond was used, and the nonbonded cutoff was set to 9 Å. The long-range electrostatics were treated with the Ewald method. These simulations provided the average density of 1.68 ± 0.04, 1.72 ± 0.04, and 1.76 ± 0.04 g/cm^3^ for 0.33, 0.50, and 0.67 mole fractions of MgCl_2_, respectively. Using the configurations corresponding to these densities, we performed MD in the NVT ensemble for 3 ns using the same thermostat. These NVT trajectories were used for analysis. It should be noted that the precise experimental densities at low T (here 585 °C/858 K) are not available in the literature; however, using the T-dependent density equation reported by Janz,^58^ we find that the computed densities are within 3.7, 3.0, and 1.2% of the extrapolated experimental densities, respectively, at 0.33, 0.50, and 0.67 mole fractions of MgCl_2_.

### Clustering Analysis

To quantify the evolving M–Cl speciation in MCl_2_–KCl mixtures where M represents either Zn^2+^ or Mg^2+^, we extracted all M–Cl-based oligomers from the NVT trajectories using a graph-theoretic^59^ framework implemented with the NetworkX^60^ library. In each frame, a bipartite M–Cl network was constructed by treating M^2+^ and Cl^–^ ions as labeled nodes, and assigning M–Cl edges whenever the interatomic distance satisfied *r*_*ij*_ ≤ *r*_cut_^M–Cl^, where *r*_cut_^M–Cl^ was chosen as the first minimum in the M–Cl partial RDF (Figures S1 and S2). The resulting M–Cl connectivity network was decomposed into connected components, each representing a chemically meaningful M–Cl cluster. Each cluster was assigned a stoichiometry (*n*_c_,*n*_a_), where *n*_c_ and *n*_a_ correspond to the number of M^2+^ cations and Cl^–^ anions in the oligomer, respectively. For every frame *f*, the anion-normalized abundance of each oligomer type, ϕ_(*n*_c_,*n*_a_)_, was computed by eq 6,

3 $$\phi_{\left(n_{c},n_{a}\right)}\left(f\right)=\frac{n_{a}\left(f\right)}{N_{{\mathrm{Cl}}^{-}}}$$

where *n*_a_(*f*) is the number of Cl^–^ ions in that oligomer. Time-averaged oligomer fractions were then obtained following eq 7,

4 $$\left\langle \phi_{\left(n_{c},n_{a}\right)}\right\rangle =\frac{1}{N_{\mathrm{frames}}}\sum_{f}\phi_{\left(n_{c},n_{a}\right)}\left(f\right)$$

yielding the overall distribution of monomers, oligomers, and extended M–Cl chains. To analyze temporal evolution and smooth short-time fluctuations, a cumulative running average was additionally applied to the time series of ϕ_(*n*_c_,*n*_a_)_(*f*).

### Free Energy Analysis

2D free energy surfaces were computed from the MD trajectories as functions of two types of cation coordination around a chloride ion. While the first one is based on the number of nearest M^2+^ ions (namely, CN_Cl_^M^), the second one describes the number of nearest K^+^ ions (namely, CN_Cl_^K^). As aforementioned, here M stands for Mg or Zn. These CNs have the same mathematical expression given below:

5 $${\mathrm{CN}}_{\mathrm{Cl}}^{M}=\sum_{i=1}^{N_{M}}\frac{1-{\left(r_{i}/r_{M}^{†}\right)}^{12}}{1-{\left(r_{i}/r_{M}^{†}\right)}^{24}}$$

6 $${\mathrm{CN}}_{\mathrm{Cl}}^{K}=\sum_{i=1}^{N_{K}}\frac{1-{\left(r_{i}/r_{K}^{†}\right)}^{12}}{1-{\left(r_{i}/r_{K}^{†}\right)}^{24}}$$

Here, *N*_M_ and *N*_K_ are the number of M^2+^ and K^+^ ions in the mixture melt. *r*_*i*_ represents the distance of the *i* ^th^ M^2+^ or K^+^ ion from a chloride ion, while *r*_M_^†^ and *r*_K_^†^ are the locations of the first minimum after the first peak in the M-Cl and Cl–K radial distribution functions (Figures S1–S4), respectively. For every chloride, CN_Cl_^M^ and CN_Cl_^K^ were calculated from the MD trajectories, which were then used to determine their joint probability distribution (*P*(CN_Cl_^M^, CN_Cl_^K^)) and the corresponding free energy surface, *W*(CN_Cl_^M^, CN_Cl_^K^) = −*k*_B_ *T* ln (*P*(CN_Cl_^M^, CN_Cl_^K^)). Here, T is the temperature and *k*_B_ is the Boltzmann constant.

## Results

Absorption results as a function of wavelength for the bismuth doped and blank 47.5% ZnCl_2_ salts at two different temperatures are shown in Figure 2, along with the bismuth doped, blank subtracted spectrum. An absorption band corresponding to Bi^3+^, consistent with the ^1^S_0_→^3^P_1_ transition, reported at ∼330 nm in literature, is clearly observed.^61,62^ Another peak is also visible in the spectra below ∼270 nm and is expected to result from the metal–metal charge transfer (MMCT) band between Bi^3+^→Zn^2+^ and/or from the ligand–metal charge transfer (LMCT) band between Cl^–^→Bi^3+^.^63,64^ There is no discernible peak location this deep in the UV regime, which prevents definitive peak assignment. Indeed, the fiber optic coupler used in the experimental setup utilizes a silver mirror that produces a significant reflectivity decrease in the UV regime, reducing the fidelity of measurement below 300 nm. However, a sudden and sharp transition in this region is expected, which reflects charge transfer that is spin- and Laporte-allowed, in contrast to the doubly forbidden ^1^S_0_→^3^P_1_ transition at slightly lower energy. The reason this forbidden transition is still relatively strong is partially due to spin–orbit coupling between the singlet and triplet states in heavy atoms like bismuth.^65^ When it is solvated in molten salt, the lack of strict symmetry in the bismuth coordination sphere and inherent disorder of the molten state at such high temperature enhance the vibronic coupling between electronic and vibrational states, causing the broadening and intensifying of spectral features.^66,67^ This is clear in the spectra shown in Figure 2b, with a more intense peak in the same salt at 400 versus 350 °C.

**Figure 2:**
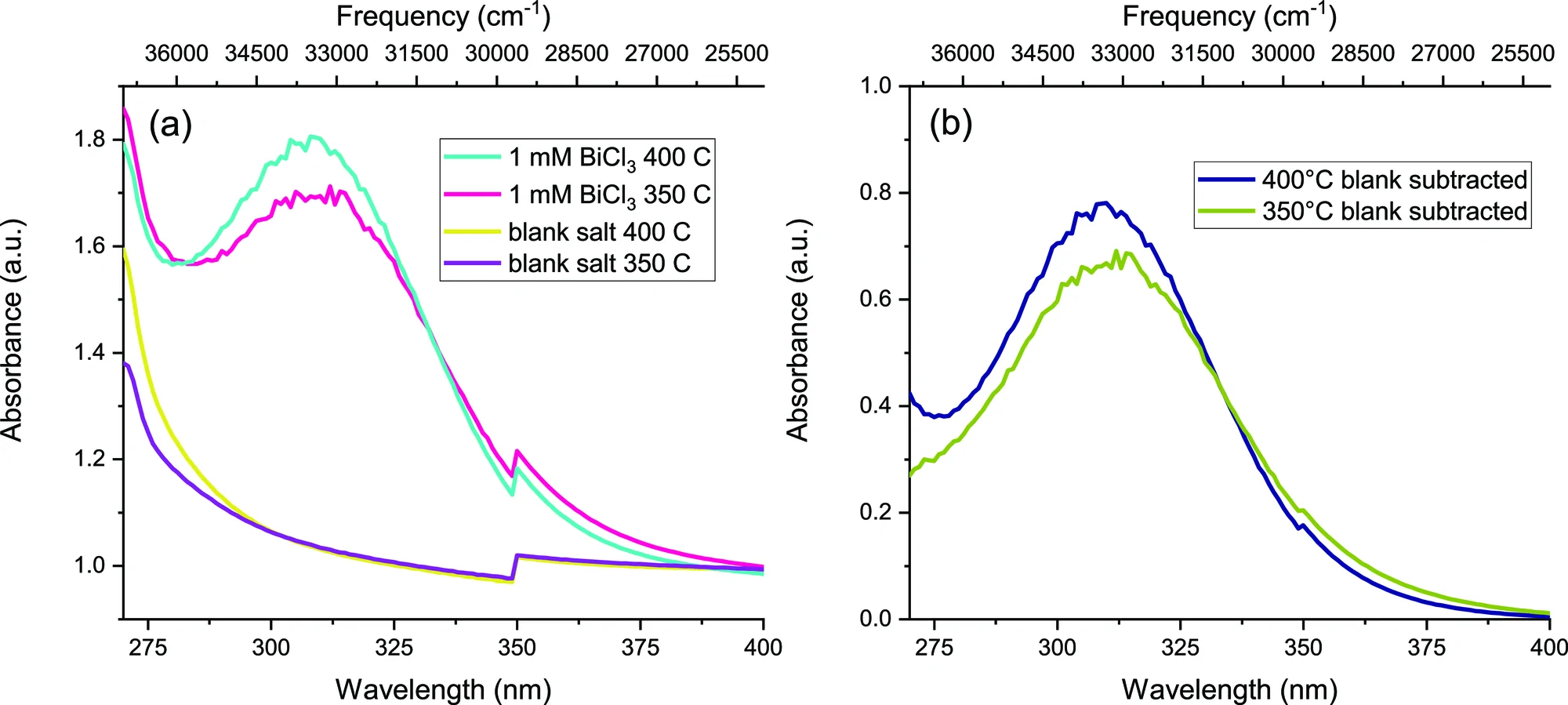
Raw spectra collected for 47.5 mol % ZnCl_2_ (balance KCl). The spectra are shown for the salt with and without 1 mM BiCl_3_ in part (a), as well as the blank subtracted, isolated Bi^3+^ spectra in part (b) for both 350 and 400 °C.

The normalized, fitted spectra for the composition-dependent bismuth absorption peak in each of the three salt systems are shown in Figure 3. For the two divalent salts, there is a clear increase in the peak maximum wavelength as a function of the amount of KCl, whereas for the monovalent salt, LiCl–KCl, the wavelength of the ^1^S_0_→^3^P_1_ transition (and thus the OB) remains relatively constant across the entire compositional range. The inset plot of Bi^3+^ in LiCl-KCl in Figure 3c more clearly shows the location of the peaks’ centroids and the lack of consistent spectral shifting with respect to composition. The OB for each salt was calculated using eq 4, using the experimentally measured peak wavelength for Bi^3+^ in LiCl-KCl at 450 °C for the reference salt, and plotted along with the accompanying frequencies as a function of composition in Figure 4. The frequencies of this peak for all of the measured salts are shown for each temperature in Table S1 as well.

**Figure 3:**
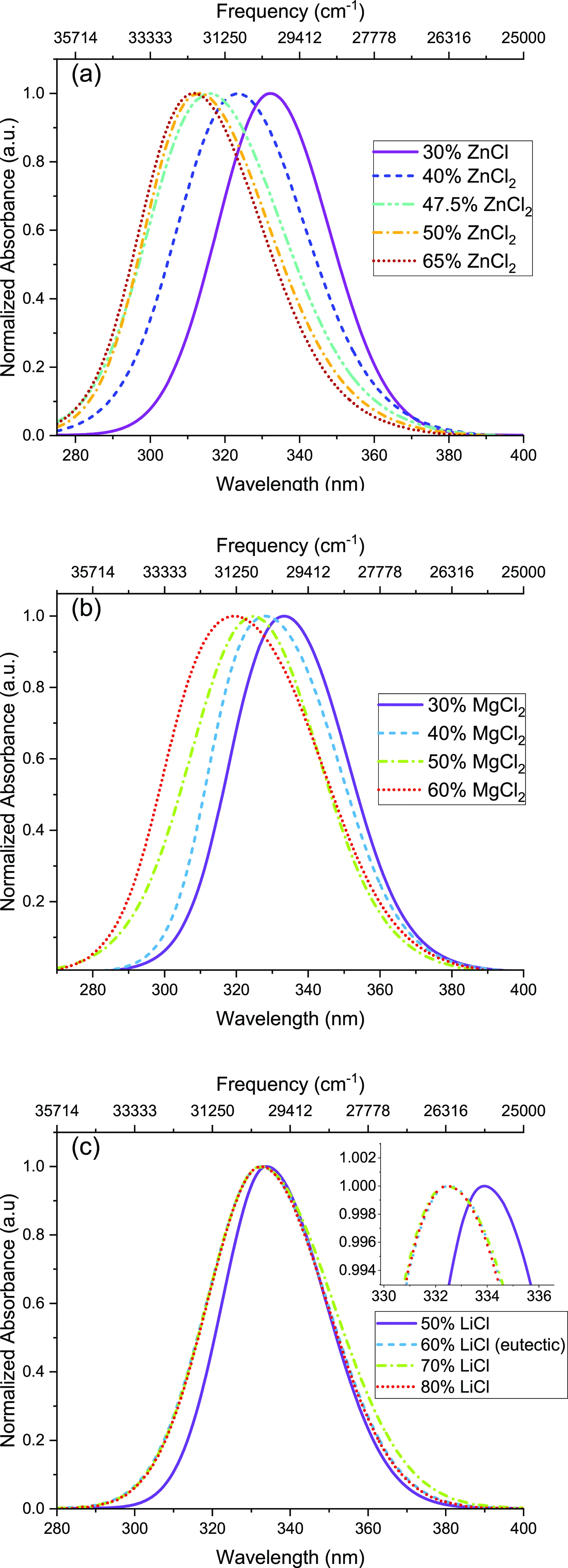
Normalized, fitted Bi^3+^ peak in (a) select ZnCl_2_–KCl salts at 450 °C, (b) MgCl_2_–KCl salts at 550 °C, and (c) LiCl-KCl salts at 550 °C. ZnCl_2_–KCl and MgCl_2_–KCl compositions >65 and 60 mol % MCl_2_, respectively, were omitted from this figure for clarity.

**Figure 4:**
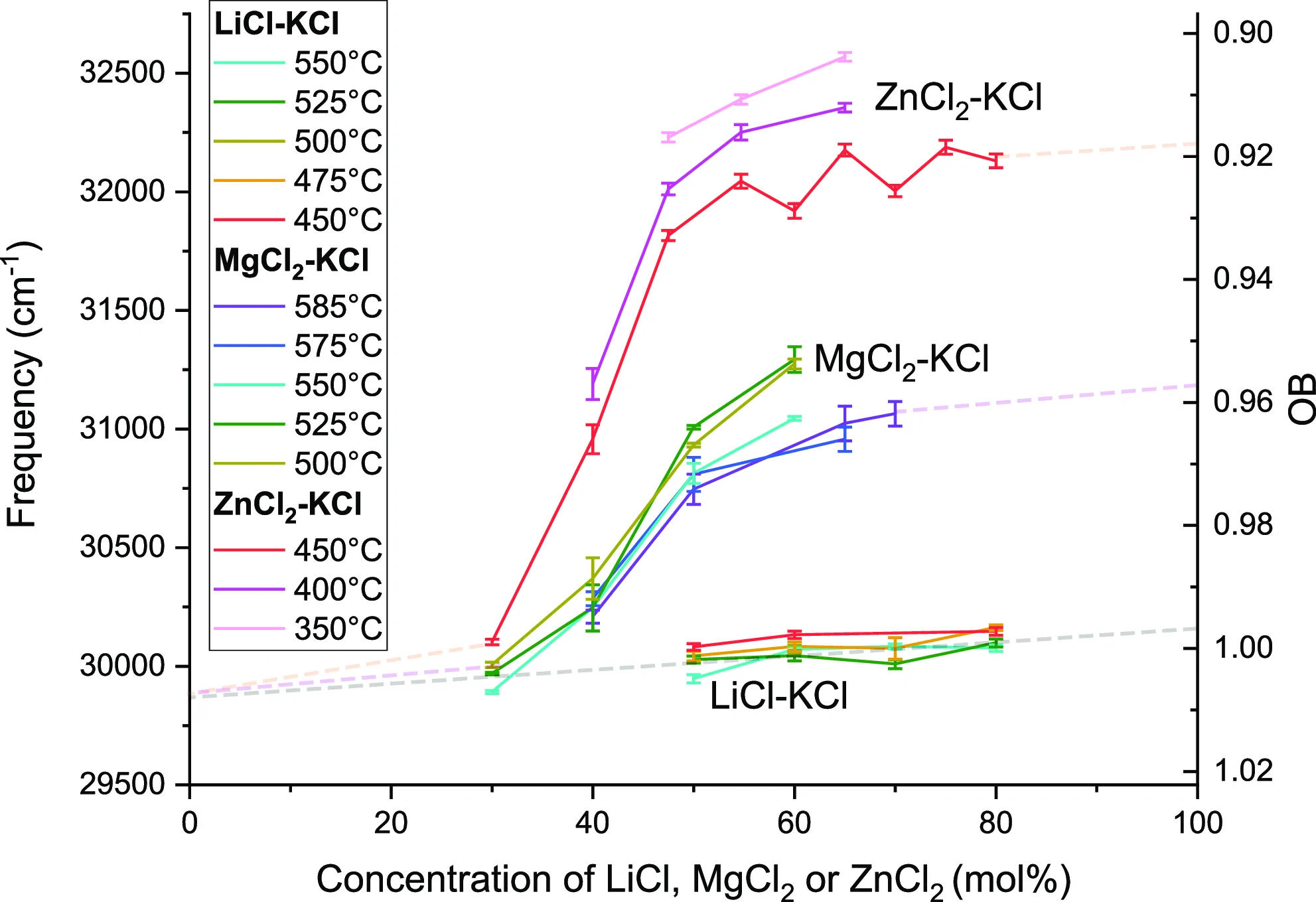
Transition energy in terms of frequency and associated OB as a function of salt composition at different temperatures. Error bars represent the error in the calculated peak maximum for each fit. The extrapolated dotted lines represent the expected basicity behavior of each salt as composition approaches 0 and 100% KCl.

The peak wavelength/basicity results for each of the three salt systems showed trends consistent with the OB theory. Optical basicity has a stronger dependence on composition in salts with multivalent constituents, such as Zn^2+^ or Mg^2+^. With the decrease in the mole fraction of ZnCl_2_ or MgCl_2_ within their respective mixtures, a corresponding decrease in bridging interactions is to be expected.^68–71^ This shifts the melt structure toward more weakly bound, nonbridging chloride, leading to higher chlorobasicity and measured OB. This trend supports the concept that salts with lower proportions of multivalent constituents tend to exhibit less polarized, and therefore more chlorobasic, environments.^55,72^

It was observed that the sensitivity of OB to composition, represented by the gradient of the curve in Figure 4, is greater in systems containing cations of higher electronegativity and, consequently, higher polarizing power. Because cation polarizing power ranks Zn^2+^ > Mg^2+^ > Li^+^, Zn^2+^ withdraws the most Cl^–^ electron density (Li^+^ the least) and produces the largest reduction in OB at a given composition. On the other hand, since LiCl and KCl are relatively more similar in cation electronegativity and chlorobasicity, there is a weak OB dependence on composition for this salt system. It is also observed that OB increases slightly with temperature. Although the origin of this behavior is more complex, one reason for this occurrence is thermal disorder, which enhances vibronic coupling in the Bi^3+^ probe and lowers the energy of the ^1^S_0_→^3^P_1_ transition. This maps to higher optical chlorobasicity in the OB framework. Another factor is that coordination numbers generally decrease with temperature, affecting the chloride density surrounding Bi^3+^.

The spread of OB values in the ZnCl_2_ salt measured using the Bi^3+^ probe is ∼0.08 over a composition range of Δ*x*_ZnCl_2__= 50 mol %, which is a relatively large scale. A steep OB gradient is observed in the 30–50% ZnCl_2_ composition range, while the OB curve flattens out as the majority of the salt becomes ZnCl_2_. A similar, albeit less obvious, trend is observed for the MgCl_2_–KCl system. The linearity in the OB gradient breaks down, and the salt OB becomes relatively constant at higher MgCl_2_ concentrations. The most likely cause for this phenomenon can be attributed to the tendency of multivalent species in a molten salt to form network-type structures with a longer range of order than the coordination sphere of the individual ionic constituents^55,73,74^ as will be elaborated upon further in the modeling results and discussion. These results do maintain consistency with historical OB work, in terms of the impact that cation electronegativity and valency have on the environment of the probe ion. The spread of basicity values is not as stratified as what would be expected in oxide systems, and this can be attributed to the lower polarizability of the chloride ion in comparison to the dinegative oxygen. Duffy and Ingram reported a decrease in basicity from 1 to 0.87 for LiCl-KCl eutectic to AlCl_3_–NaCl (67:33).^29^ For comparison, we see a decrease in OB from 1 to 0.96 for LiCl-KCl to MgCl_2_–KCl (65:35). It is to be expected that a trivalent cation like Al^3+^ would have an even greater impact on basicity than divalent Mg^2+^.

To complement the experimental OB measurements, we quantified how Zn_n_Cl_m_^(2n–m)^ and Mg_n_Cl_m_^(2n–m)^ structural motifs evolve with composition in MCl_2_–KCl melts. Figure 5a,c summarizes their average populations across the compositional range for ZnCl_2_–KCl and MgCl_2_–KCl, respectively.

**Figure 5:**
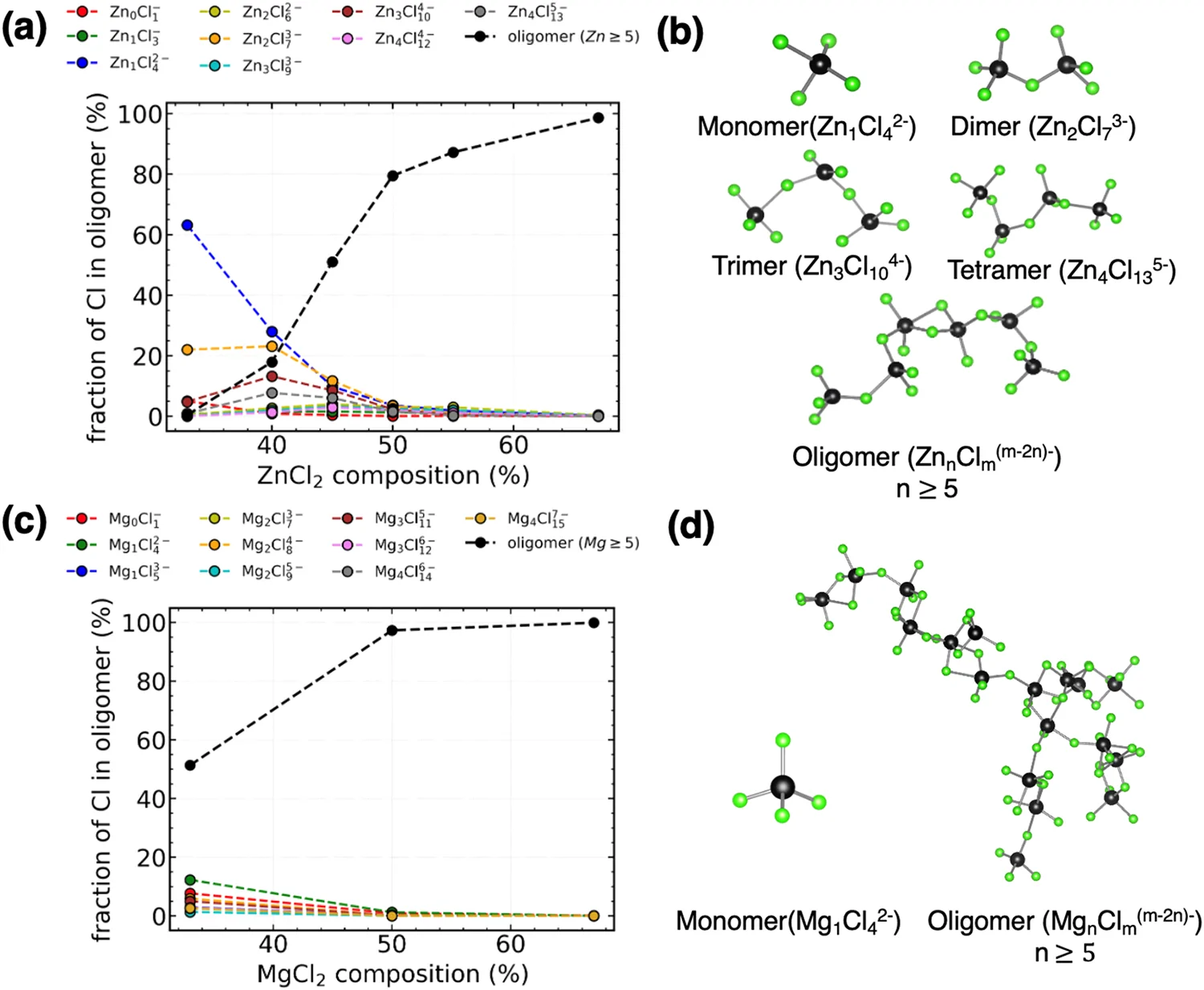
Speciation as a function of (a) ZnCl_2_ and (c) MgCl_2_ compositions, illustrating the rapid growth of oligomeric M^2+^–Cl^–^ chains from 33 to 50 mol % MCl_2_ and a more gradual attenuation of oligomeric species above 50 mol % MCl_2_, approaching nearly 100%. The most important structural motifs of (b) Zn_n_Cl_m_^(m–2n)–^ (d) Mg_n_Cl_m_^(m–2n)–^ observed in molten MCl_2_, ranging from isolated MCl_4_^2–^ (monomer) units to higher-order network-like aggregates.

At 33 mol % ZnCl_2_, Zn^2+^ is predominantly present in small units (monomers and dimers). No extended chains were detected at this composition. Short chains begin to form at 40–45 mol % ZnCl_2_, as reflected by the appearance of large clusters (defined here as clusters containing ≥5 Zn^2+^). A substantial increase in chain-containing species is observed at 50 mol % ZnCl_2_, where more than 80% of Cl^–^ ions participate in extended chain-like structures. This growth continues at 55 and 67 mol % ZnCl_2_, where Zn–Cl chain-containing species become increasingly prevalent, and the melt approaches a fully connected ionic network. For a MgCl_2_–KCl mixture with the same mol % as that of the ZnCl_2_–KCl mixture, relatively larger Mg–Cl species are formed (Figure 5b). In fact, an almost fully connected ionic network can already be seen at 50 mol % MgCl_2_ (Figure 5d).

One interesting finding from Figure 5 is that the fraction of Cl^–^ that is not bound to Zn^2+^ or Mg^2+^ is significant. We find that approximately 5 and 10% of Cl^–^ at 33 mol % MCl_2_ are not bound to Zn^2+^ and Mg^2+^, respectively. These Cl^–^ ions are bound to K^+^ ions. We discuss below, in terms of free energy landscapes, that no Cl^–^ is truly free; rather, all are bound to at least one of these cations.

Figure 6 depicts 2D free energy surfaces that illustrate how the magnesium and zinc coordination numbers (CN_Cl_^Mg^ and CN_Cl_^Zn^) of a chloride ion correlate with its potassium coordination number (CN_Cl_^K^) as the MgCl_2_ or ZnCl_2_ mol % varies. The absence of a free energy minimum corresponding to (CN_Cl_^Mg^ = 0, CN_Cl_^K^ = 0) and (CN_Cl_^Zn^ = 0, CN_Cl_^K^ = 0) confirms that Cl^–^ must be cation-bound—whether exclusively to a specific type or simultaneously to different types. Among the many possible cationic environments around a chloride ion, Figure 6 highlights several key snapshots in which the relative likelihood changes with salt composition. For instance, the common cationic environment for both systems at 67 mol % (i.e., a Zn- or Mg-rich environment) is the coordination of a Cl^–^ ion with two Mg^2+^ (or Zn^2+^) and one or two K^+^ ions. While a Cl^–^ ion can coordinate with three Mg^2+^ ions, coordination with three Zn^2+^ ions is prohibited. In a Mg/Zn-low environment (33 mol %), a Cl^–^ ion is likely surrounded by one Mg^2+^ or Zn^2+^ and four K^+^ ions. Finally, what stands out most strikingly is the region of the free energy surface describing zero Mg^2+^ or Zn^2+^ coordination with a Cl^–^ ion. While at 67 mol % there are no Cl^–^ ions exclusively bound to K^+^ ions in either system, a state with 4–5 K^+^ ions coordinating with a Cl^–^ exists at 50 mol % MgCl_2_ but not at 50 mol % ZnCl_2_. Reducing the mol % further to 33%, this state becomes more prominent (deeper, bluish minimum) for MgCl_2_ and finally appears for ZnCl_2_ with reduced likelihood (greenish minimum).

**Figure 6:**
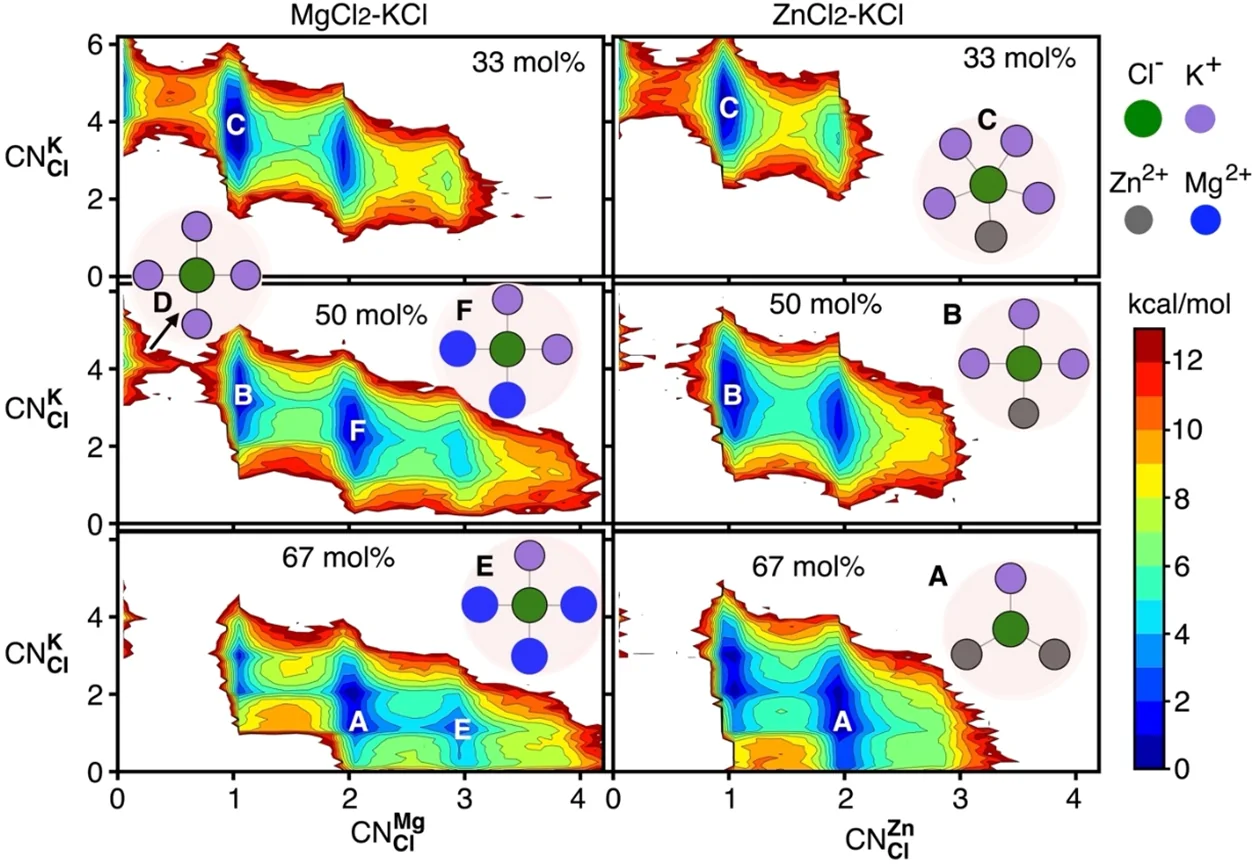
2D free energy surfaces correlating K coordination number of Cl^+^ to its Mg^2+^ (left) and Zn^2+^ (right) coordination numbers, respectively, in the MgCl_2_–KCl and ZnCl_2_–KCl mixtures. Different possible cationic coordination environments around the Cl^–^ ion are highlighted with A–F; the environments A–C, highlighting Zn^2+^ coordination, are used interchangeably for showcasing Mg^2+^ coordination as well.

The greater occurrence of Cl^–^ ions bound only to K^+^ in MgCl_2_–KCl mixtures than in ZnCl_2_–KCl mixtures suggests that it is easier to remove chlorides from their Mg^2+^-bound states than from their Zn^2+^-bound states. To validate this, we converted the Mg–Cl and Zn–Cl radial distribution functions into free energy profiles, defined as *W*(*r*) = −*k*_B_ *T* ln­[*g*(*r*)], where *g*(*r*) is the radial distribution function and *W*(*r*) is the associated free energy. In Figure 7, we depict these free energy profiles normalized by the thermal energy (*k*_B_ *T*), alongside the Boltzmann probability of removing a Cl^–^ ion from the first coordination shell of a Mg^2+^ or Zn^2+^ ion, calculated using the corresponding free energy barrier, i.e., the height from the first minimum to the first barrier maximum. Across all compositions—from Mg/Zn-low to Mg/Zn-rich environments—removing a Cl^–^ ion is energetically more favorable from its Mg^2+^-bound state than from its Zn^2+^-bound state. It should be recognized that the elevated temperature of the MgCl_2_–KCl systems facilitates this higher Cl^–^ removal probability from the Mg-bound state.

**Figure 7:**
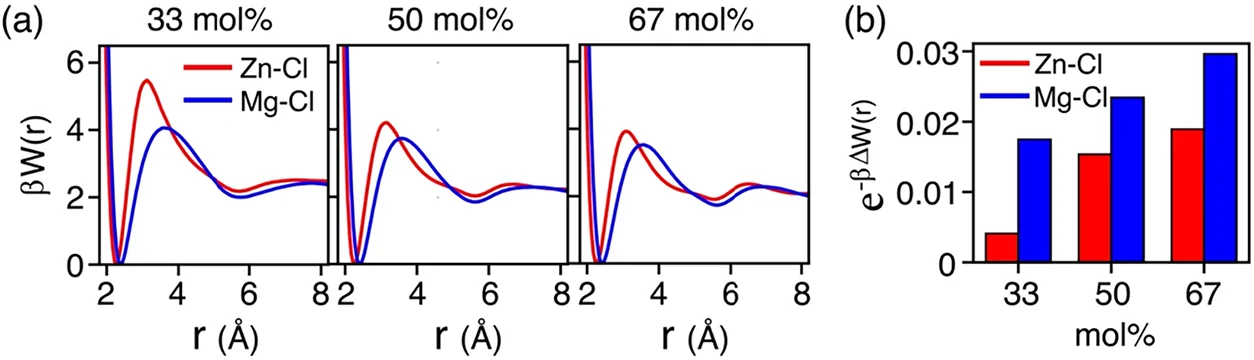
At different MgCl_2_ (ZnCl_2_) mol %, (a) normalized free energy profile of the Zn–Cl (Mg–Cl) distance and (b) the Boltzmann probability of removing a Cl^–^ ion from the first coordination shell of a Zn^2+^ (Mg^2+^) ion. β is the inverse thermal energy, 1/*k*_B_ *T*.

## Discussion

The OB trends compared across X-KCl systems (X = MgCl_2_, ZnCl_2_, and LiCl) tell a compelling story of how cation identity influences the electronic environment of molten salt systems. The more electronegative cations withdraw more Cl^–^ electron density, generally resulting in a less basic salt at a given X:KCl ratio. Optical basicity pioneers Ingram and Duffy had coined a definition for the parameter as the average polarization of a probe ion’s surroundings.^29,75,76^ In other words, OB reports the amount of charge density donated to the probe by surrounding anions. Several factors contribute to the chlorobasicity of a salt composition, including the polarization power and oxidation state of the cations, as well as the stoichiometric ratio of cations to anions. The effects will be sorted into two categories for the purpose of this discussion: cation identity and binary salt composition in terms of % KCl.

### Hierarchy of Cation Influence on Basicity

Upon close examination of salts at equivalent compositions, a clear hierarchy emerges. LiCl-KCl mixtures consistently exhibit the highest OB values, followed by MgCl_2_–KCl, with ZnCl_2_–KCl showing the lowest basicity at any given composition. This ordering directly reflects the polarizing power of each cation species and their ability to withdraw electron density from surrounding chloride ions. More electronegative cations create a more electron-deficient environment around the chloride ions, resulting in lower OB values. The progression from Li^+^ to Mg^2+^ to Zn^2+^ represents an increase in electronegativity and polarizing power, for which the impact on OB is clearly depicted in Figure 4. The lower charge density of Li^+^, compared to divalent Mg^2+^ and Zn^2+^, results in the high OB of its completely monovalent mixtures with KCl. Both lithium and potassium chloride act as ionic, ‘network-breaking’ salts;^73,74,77–81^ thus, significant changes in polarizing power on either side of the compositional space are not expected. This is the primary reason eutectic LiCl-KCl is used as a high-basicity reference salt on the OB scale.

Fundamental differences in electronic character between Li^+^ and Zn^2+^ chlorides have been revealed by Nguyen et al. through first-principles molecular dynamics.^82^ It was observed that Cl^–^ behaves more ionically (has higher local electron density) in LiCl-KCl eutectic compared to pure ZnCl_2_, as demonstrated by the calculated average valence electron occupancies (Bader charges; ∼7.86 e^–^ for LiCl-KCl and ∼7.55 e^–^ for ZnCl_2_). The higher electron density of chloride in LiCl-KCl is available to share with Bi^3+^ probe ions. In contrast, chloride electron density in ZnCl_2_ is reduced by covalent Cl–Zn interactions, forming an extended molecular orbital network bridged with unidirectional, sigma-type bonds between p and d orbitals of Cl^–^ and Zn^2+^, respectively. In contrast, there is no significant overlap between Li^+^ or K^+^ and Cl^–^ orbitals, indicating that the polarization occurring between the anions and cations in LiCl-KCl melts is ionic in nature. This prior work by Nguyen et al.^82^ also explored the impact of chlorobasicity on the behavior of the ZnCl_2_ and LiCl-KCl salts under irradiation, which is inevitable during operation of a molten salt reactor. In LiCl-KCl, which acts as a Lewis base, dissolved Cl_2_ gas formed as a product of radical recombination behaves as an electron acceptor to form Cl_3_^–^ via disproportionation reaction, while Cl_2_ remains as-is in acidic ZnCl_2_. The higher solubility of Cl_2_ gas in alkali halides compared to ZnCl_2_ and MgCl_2_ observed by Andresen et al.^83^ is consistent with stabilization of dissolved chlorine as Cl_3_^–^ in alkali halide melts.^82^

In addition to electronic character, cation size, which is inversely related to electronegativity, has also been shown to play a significant role in the local structure and degree of ionicity in molten salts.^78,84–86^ However, this effect alone does not effectively explain the OB data presented here. Indeed, the species in this work vary marginally in ionic radii (Mg^2+^ and Zn^2+^ have radii of 0.66 and 0.60 Å for their expected coordination numbers of 5–6 and 4, respectively) and Li^+^ is even smaller (r = 0.59 Å for CN = 4) than Mg^2+^ and Zn^2+^.^87^

While Mg^2+^ is more charge-dense than Zn^2+^,^88^ the latter is significantly more polarizing due to its electronic configuration. While Mg^2+^ is isoelectronic with Ne, Zn^2+^ has a full outer 3d^10^ orbital, which gives it pseudonoble gas character,^89,90^ i.e., anomalously higher tendency toward covalent bonding.^89–91^ There is poorer shielding of electrons in the d orbital that results in a greater pull toward the positively charged nucleus, a smaller than predicted ionic radius, and more covalent bonding character.^91^ The effect of cation electronegativity on anion polarizability and resulting OB has been previously investigated for molten oxide glass systems and follows similar trends as the ones observed in this study.^17,28,92–96^

Herein, from free energy surfaces (Figures 6 and 7), we can clearly distinguish between the chlorobasicity of MgCl_2_–KCl and ZnCl_2_–KCl melts. We find from the 2D surfaces that chlorides are never truly “free” in these melts—they are bound to exclusively either cations or shared by them. Across moderate-to-low divalent salt concentrations, MgCl_2_–KCl develops a distinct low-energy basin for K^+^-only coordination around chlorides, which is much less prevalent for the ZnCl_2_–KCl melts. This difference arises from the higher free energy barriers and, consequently, lower dissociation probabilities of Zn–Cl bonds relative to Mg–Cl bonds (Figure 7). Such energetically favorable chloride sequestration by Zn^2+^ limits the electron density available for donation to the Bi^3+^ probe, providing a direct microscopic basis for the lower chlorobasicity of ZnCl_2_–KCl compared to MgCl_2_–KCl.

### Compositional Dependence and Structural Origins of OB Nonlinearity

Perhaps the most intriguing aspect of these findings lies not just in the absolute basicity values of each salt at a fixed %KCl, but in how these values change with composition. The slopes of the basicity curves reveal fundamental differences in how each salt system responds to compositional changes. It is not sufficient to consider salt’s chlorobasicity as a product of charge-neutralization, as the residual charge is not necessarily linear with respect to the stoichiometry of a multicomponent salt composition.

In the complete absence of covalency, the theoretical OB of a binary molten salt would follow a simple rule of mixture (see eq 10), as a weighted sum of the OB of each pure component.^96^

7 $$\Lambda =x_{a}\Lambda_{a}+x_{b}\Lambda_{b}$$

The linearity and small slope of the LiCl-KCl basicity curve are reflective of the similarities between lithium and potassium chloride molten salts. Neither cation significantly alters the local electronic environment upon mixing due to their similar and relatively low polarizing powers (in comparison to Zn^2+^ and Mg^2+^).^72^ However, for salts that form networks consisting of bridging chlorides, the basicity deviates from a linear mixing rule, suggesting that compositional changes alter the local chloride electronic environment in ways not captured by a simple weighted average.^92,93^

Both divalent salts exhibited nonlinearity in the OB gradient as a function of composition to some extent. Figure 4 shows that ZnCl_2_–KCl salts converge more sharply into a constant basicity value than the MgCl_2_–KCl salts. However, data for MgCl_2_ compositions greater than 70 mol % MgCl_2_ are limited due to the prohibitively high melting points and limitations of the experimental setup. Both divalent salts exhibit relatively linear OB dependence on composition when KCl is the major component, which is attributed to increased ionicity and the breakage of intermediate-range order brought on by the introduction of a “network-breaking” or “spacer” salt.^55,71,97^

This observed ionic nonideality can be justified from the oligomer analysis results (Figure 5) obtained from MD simulations of MCl_2_–KCl mixtures. The experimental basicity decreases sharply between 33 and 50 mol % ZnCl_2_, after which the slope abruptly flattens. The MD results identify the same compositional interval as the point where the melt transitions from a predominantly monomeric/dimeric (33 mol %) speciation to the one dominated by extended Zn–Cl–Zn chains (50 mol %). As shown in Figure 5a, below ∼40 mol % ZnCl_2_, the melt contains mainly small clusters with predominance of monomer to tetramer. As the composition approaches 45 mol % ZnCl_2_, larger chains with Zn ≥ 5 start to appear (∼50% of the total Cl^–^), indicating the onset of network formation. By 50 mol % ZnCl_2_, the system undergoes a clear structural transition with more than 80% of Cl^–^ anions incorporated into chain-like motifs. Above 50 mol % ZnCl_2_ this growth in larger species continues, and by 67 mol % nearly all Cl^–^ ions reside in extended chains, leaving only a small fraction in discrete oligomers. In this extended network-dominated regime, further increases in ZnCl_2_ primarily lengthen existing networks rather than create additional terminal chloride sites, which naturally explains the weak composition dependence of the measured OB at high ZnCl_2_ content. For MgCl_2_–KCl systems, the trend is similar; extended connected networks start to form even at relatively lower (50 mol %) MgCl_2_ concentration.

This emergence of network connectivity explains the abrupt change in OB behavior measured experimentally. In the low-MCl_2_ regime, each incremental addition of MCl_2_ increases the population of bridging chloride sites, which substantially lowers the extent of electron-donation to the Bi^3+^ probe and produces the steep OB gradient. Once the network is established, however, the fraction of bridging chloride saturates, and any additional MCl_2_ then increases M-Cl chain lengths but does not significantly alter the average ionic environment, yielding the observed plateau in OB at higher MCl_2_ content. Thus, MD simulations demonstrate that the nonlinear composition dependence of OB originates from the formation and saturation of M–Cl–M chain networks.

A stronger decrease in basicity in ZnCl_2_–KCl than in MgCl_2_–KCl is also mirrored by the lower population of Cl^–^ ions not coordinated to the divalent cation (Figure 6), pointing to stronger retention of chloride by Zn-centered species. Consistent with this, Zn-containing melts favor shorter clusters with a higher fraction of terminal chloride, whereas Mg-containing melts retain a larger population of labile bridging Mg–Cl–Mg species that are more accessible for coordination with Bi^3+^.

It is worth noting that this network-driven interpretation is consistent with the classical polarizable ion modeling. Utilizing early PIMs to calculate structure factors (calculated directly from simulated ion positions rather than from Fourier transformed radial distribution functions within a finite box as to avoid error) for a series of divalent molten chloride salts, Madden et al. reported that as cation M^2+^ size decreases, molten MCl_2_ radial distribution functions (RDFs) depart more strongly from simple rigid ion models (the Born–Mayer model), which neglect polarization effects.^98,99^ More relevantly, for cations in the size range of Zn^2+^, the calculated RDFs suggest the existence of local tetrahedral coordination. Mg^2+^ is of similar size to Zn^2+^; however, the primary coordination number of MgCl_2_-rich melts tends toward 5.^71^ The structure factor calculations reveal a low-Q prepeak for the medium-sized cation melts (not seen with larger cations). This feature corresponds to correlations on a length scale about twice the nearest cation–cation distance, i.e., intermediate-range ordering.^55,71,73,78,79^ This suggests that highly polarizable melts favor corner sharing between neighboring tetrahedral units and the formation of a network of bridges by Cl^–^ anions in the intermediate size range.

Polarizable anions tend to form nonlinear bridges between polarizing cation pairs because the induced dipole from the polarizing cations occurs along the M-Cl-M plane in such a way that a negative charge is forced along the centers between the two cations. In these MCl_2_ melts, Cl^–^ anions screen the cation–cation Coulomb repulsion, weakening the effective interaction and stabilizing shorter cation–cation separations.^71,100–102^ The corner sharing as described above transitions to edge sharing as the cation size decreases, leading to the formation of four-membered rings comprised of two chloride bridges as depicted in Figure 8. It has been suggested that in pure ZnCl_2_ melts at 600 K, at least 20% of the Zn tetrahedra exist within edge-sharing networks, which decrease in length with an increased temperature.^103–105^ The addition of network-breaking cations also decreases the fraction of edge sharing and increases the amount of nonbridging chlorides,^106^ increasing the chlorobasicity of the salt. This has been seen in spectroscopic studies of NiCl_2_ dissolved in ZnCl_2_–KCl melts.^107^ The dilute (2 mol %) Ni­(II) does not significantly perturb the melt; rather, it is incorporated into the Zn–Cl network. Increasing the KCl concentration decreased the number of chloride-shared interactions and multimeric chains. This is supported by the finding that increasing the KCl concentration decreases the population of five-coordinate and increases that of the four-coordinate Ni­(II). This is also consistent with the increased population of tetrahedral Ni with increased % KCl. Increasing the temperature further reduced the populations of five- and six-coordinate Ni­(II).

**Figure 8:**
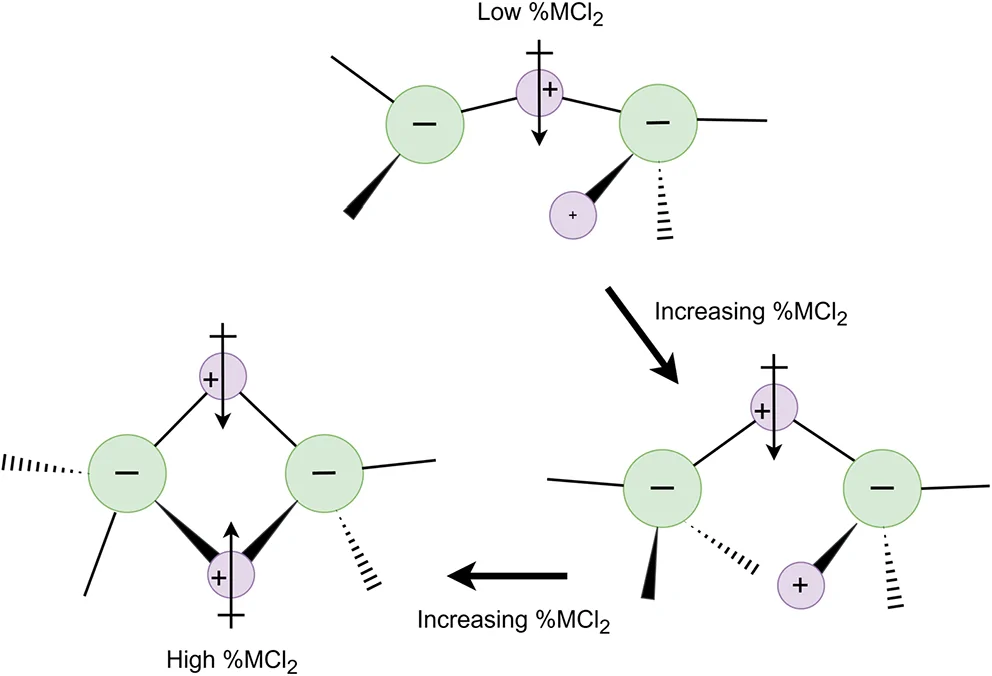
Schematic of the formation of edge-sharing rings in an MCl_n_ molten salt, adapted from Madden and Wilson with permission,^100^ © 2001 IOP Publishing. Cl^–^ and M^n+^ ions are represented by the large green and small purple circles, respectively. Bonds without terminal atoms pictured represent availability for ligands in the metal center’s coordination sphere. Increased edge sharing is correlated with decreased chlorobasicity.

The structure of the MgCl_2_–KCl system was also investigated in a 1980 Raman study by Brooker and Huang.^108^ In melts with high MgCl_2_ content, the Raman spectrum began to show an additional peak previously misinterpreted as representing the Mg_2_Cl_7_^3–^ dimeric ion. However, recent work combining *ab initio* MD simulations with experimental Raman spectroscopy has demonstrated that the five-coordinate MgCl_5_^3–^ complex is the dominant species in pure MgCl_2_ rather than a dimeric species.^71^ The peak that had incorrectly been suspected to represent an edge-sharing dimer emerges with increased MgCl_2_ concentration due to changes in coordination equilibria. In general, Mg^2+^ maintains composition-dependent equilibrium between several possible coordination states in the molten state—at high MgCl_2_ concentrations, five-coordinate MgCl_5_^3–^ polyhedra with distorted square pyramidal geometry predominate, while four-coordinate tetrahedral MgCl_4_^2–^ units become increasingly more stable as KCl concentration increases. This is reinforced by the formation of extended networks of MgCl_5_^3–^ units in pure MgCl_2_, connected by corner- (∼70%) and edge-sharing (∼30%) chlorides. The density and length of these networks diminish with the addition of KCl, which is coincident with the decrease in coordination of Mg^2+^ from predominantly five to a more even distribution between five and four at 50 mol % of MgCl _2_.^62,106^

Further evidence is provided in a recent study by Wu et al.^74^ in which four-coordinate MgCl_4_^2–^ was the most stable state at 15 mol % MgCl_2_, distorted square pyramidal geometry (CN = 5) was more stable at 50 mol % MgCl_2_, and the population of a fully octahedral complex (CN = 6) increased with increasing MgCl_2_ concentration. In other words, increasing the amount of KCl in the salt decreases the coordination number of Mg^2+^, which is associated with the decomposition of polynuclear (MgCl_2_)_n_ chains and the formation of a more random melt structure with discrete MgCl_4_^2–^ tetrahedra. Consistent with this, the MD cluster analysis (Figure 5c) shows that MgCl_4_^2–^ monomers are the most prevalent nonoligomeric species at 33 mol % MgCl_2_.

The MD simulations in the current work are consistent with the network disruption picture painted by Roy et al.^71^ and Wu et al.,^74^ while adding further compositional resolution. As shown in Figure 5c,d, the fraction of Cl^–^ involved in large Mg–Cl oligomers (*n* ≥ 5) remains high across a wide compositional range, approaching ∼50% even at 33 mol % MgCl_2_ and reaching nearly 100% at 50 mol % MgCl_2_. It is important to note that the cluster analysis employed here, which tracks the fraction of Cl^–^ participating in oligomers of any size above a threshold, is a different metric from the intermediate-range order and network connectivity analysis used by Roy et al. and Wu et al., which characterized the disruption of networks relative to pure MgCl_2_. These descriptions reflect complementary aspects of the same structural changes. KCl does progressively disrupt the extended MgCl_5_^3–^ networks of pure MgCl_2_, reducing their density and length, while a significant fraction of Cl^–^ nonetheless remains associated with oligomeric Mg–Cl structures even at lower MgCl_2_ concentrations. Compared to ZnCl_2_–KCl, where its transition from negligibly to predominantly oligomeric occurs sharply between 33 and 50 mol % ZnCl_2_, the MgCl_2_–KCl system shows an earlier and more gradual buildup of oligomeric species—consistent with the tendency of Mg^2+^ toward higher and more variable coordination numbers relative to the rigidly tetrahedral Zn^2+^. Approximately 10% of Cl^–^ is not directly bound to Mg^2+^ at 33 mol % MgCl_2_, compared to ∼5% unbound from Zn^2+^ at the equivalent composition, reflecting the greater ease with which Cl^–^ can be removed from Mg^2+^ coordination—a point further supported by the free energy analysis of Figures 6 and 7.

### Compositional Extremes

It is pertinent to note that in Figure 4, linearly extrapolating the basicity curves for the MgCl_2_ and ZnCl_2_ salts would result in an OB that is impossibly large for a 100% KCl molten salt. Based on the data presented for the LiCl-KCl system and the assumed high ionicity of this melt across the entire compositional regime, extrapolation to %LiCl = 0 should provide a reasonable estimated OB value for 100% KCl. Consequently, the MgCl_2_ and ZnCl_2_ curves must taper at high KCl concentration to this value, resulting in a shape reminiscent of a traditional acid–base titration curve. This behavior closely reflects what had been observed using Bi^3+^ and Pb^2+^ as OB probes in the molten AlCl_3_–KCl system.^109,110^ In the works of Angell and Bennett, there is a clear “equivalence point” in this binary salt separating the regions of saturated acidity and basicity created by the stark difference in Cl^–^ polarizability between AlCl_3_ and KCl. The same logic may be extended to the salt systems in the present work, with the exception that Mg^2+^ and Zn^2+^ form more extended aggregates that grow in size as their content increases. The most polarizing cation, Zn, seems to saturate in acidity more drastically than Mg and Li. In other words, as the polarizing power of the cations in the salt decreases, the shape of the basicity curve may approach linearity.

### Temperature Dependence

The temperature dependence shown in Figure 4 adds another layer to this discussion. While all systems show a general increase in OB with increasing temperature, the magnitude of these changes varies with the cation character. At the ionic extreme, LiCl-KCl shows minimal temperature dependence, while the more covalent ZnCl_2_–KCl system exhibits greater sensitivity to temperature changes, consistent with the thermal disruption of polarization-induced structural features, which has been well described by Wu et al.^74^

While there is limited data regarding chlorobasicity measurements via OB and even fewer that investigate temperature effects,^2,29,31,109,111,112^ the present work does agree with the temperature dependence findings in the work of Sharpless et al., in that melts composed of more polarizing cations, i.e., Al^3+^ in their work and Zn^2+^ in ours, have OBs that increase more drastically with a given temperature increase. There exists a more well-established connection between the OB of a melt and its ionic conductivity,^24,25,113^ which is known to be a temperature-dependent parameter. Increasing the OB of a melt typically increases its ionic conductivity linearly. While the observed correlation between OB and electrical conductivity does not establish causation, the consistent temperature dependence of both properties suggests that they may respond similarly to thermal effects on melt structure. Of the works that investigate this dependence, a positive correlation between the OB and temperature is reported.^110,112,114–116^

However, it can be argued that the temperature has contradictory effects on chlorobasicity. There is increased molecular motion within a melt with increasing temperature, resulting in the distortion of coordination environments to favor lower oxidation states, as has been observed for Cr, Eu, and Cu in molten silicate melts.^117–119^ This is particularly relevant because it has been shown that the OB of silicate melts is proportional to the amount of higher oxidation-state species for nearly all redox couples studied.^10^ This relationship is attributed to the tendency for specific coordination states to be stabilized by specific, compositionally dependent melt structures.^120^ Similarly, Fe and Co have been shown to change their coordination from octahedral to tetrahedral upon increasing basicity.^115^ This is coincident with the previously described increased stability of higher-coordinate (CN = 5 and 6) Mg-centered polyhedra with high MgCl_2_ concentration, i.e., low chlorobasicity. In another study by Gill et al., the M-Cl coordination number for dissolved NiCl_2_ and CoCl_2_ in pure ZnCl_2_ tended to decrease with increasing temperature.^121^ It can be argued that the increased entropy at higher temperatures favors less coordinated complexes.

These results show that higher temperature affects the melt structure and metal speciation in the same way as lower basicity does. This presents a complex relationship where temperature not only affects the spectroscopic measurement of OB through thermal motion but also influences the very structural parameters that OB is often employed to characterize. The result is that temperature effects on molten salt systems cannot be understood through an overall increase in thermal energy alone. Rather, careful consideration is required of how temperature-induced structural changes interplay with the fundamental acid–base properties of the melt.

## Conclusions

This study demonstrates the ability of Bi^3+^ as an OB probe ion to successfully respond to fundamental differences in electronic environments across three molten chloride systems. A clear hierarchy emerges where LiCl-KCl exhibits the highest OB, followed by MgCl_2_–KCl, with ZnCl_2_–KCl showing the lowest values, reflecting the progression of cation polarizing power, as well as how electronic and structural properties drive both absolute basicity values and their compositional dependencies. This is not to say that Bi^3+^ is universally effective as a probe ion since there are observations of probe ion site-selectivity in highly basic^29^ or acidic^109^ and structurally heterogeneous melts.^122^ Selection of a probe ion should be made in consideration of the cations constituting the salt being measured. Additional studies of Pb^2+^ or Tl^+^ as a probe for the three systems investigated for this work would confirm the efficacy of each probe in relation to the others. It is also pertinent to note that BiCl_3_ (boiling point = 447 °C) considerably volatilizes at temperatures >450 °C, making OB measurements of salts with very high melting points more difficult.

The compositional dependencies observed here do reveal distinct structural responses: LiCl-KCl follows near-linear behavior consistent with ideal ionic mixing, while both divalent systems exhibit pronounced nonlinearity, characteristic of an acid–base titration curve. This deviation signals the formation of chloride-bridged networks, with ZnCl_2_ showing more definitive tetrahedral coordination compared to the structurally diverse coordination states of MgCl_2_. The network-forming behavior of salts containing ZnCl_2_ and MgCl_2_ was validated computationally by using oligomer analysis techniques enabled by MD simulations. Temperature effects add complexity, where thermal disruption competes with the spectroscopic responses to molecular motion.

These findings establish OB as a sensitive parameter for understanding molten salt systems at a molecular level and provide insights into predicting and controlling the chemical behavior of complex molten salt systems in practical applications in the nuclear energy sector and beyond. A thorough understanding of molten salt chlorobasicity and its dependencies may enable the design and selection of salt compositions that minimize the degradation of structural materials used in molten salt reactors and thermal storage tanks.

## Supplementary Material



## Data Availability

The digital data for all figures, tables, charts, and any other media contained in this publication and its associated files will be made accessible in the MSEE Community of the Zenodo repository, under Digital Object Identifier (DOI): 10.5281/zenodo.22212000.

## Notes

UV Vis chlorobasicity (PDF)
