# Supplementary material for: In Situ UV–vis Quantification of Chlorobasicity in Binary Molten Chlorides
Source: J Phys Chem B. 2026 Aug 28;130(40):10719–34. doi: 10.1021/acs.jpcb.6c03131 (PMC13656735; doi:10.1021/acs.jpcb.6c03131)
Supplement: Supplementary file 1 [file jp6c03131_si_001.pdf]

In-Situ UV–Vis Quantification of Chlorobasicity in Binary Molten Chlorides

Supporting Information

Kailee Buttice[a], Shubhojit Banerjee[b], Thiago J. Pinheiro dos Santos[b], Ellie Kim[c], Qiufeng Yang[d], Alejandro Ramos-Ballesteros[d], Sheng Dai[b,c], Ruchi Gakhar[d], Santanu Roy[b], Vyacheslav S. Bryantsev*[b], Adrien Couet*[a]*

[a]Department of Nuclear Engineering and Engineering Physics, University of Wisconsin-Madison, Madison, WI 53706, United States

[b]Chemical Sciences Division, Oak Ridge National Laboratory, Oak Ridge, TN 37830, United States

[c]Department of Chemistry, University of Tennessee, Knoxville, TN 37916, United States

[d]Advanced Technology of Molten Salts, Idaho National Laboratory, Idaho Falls, ID 83415, United States

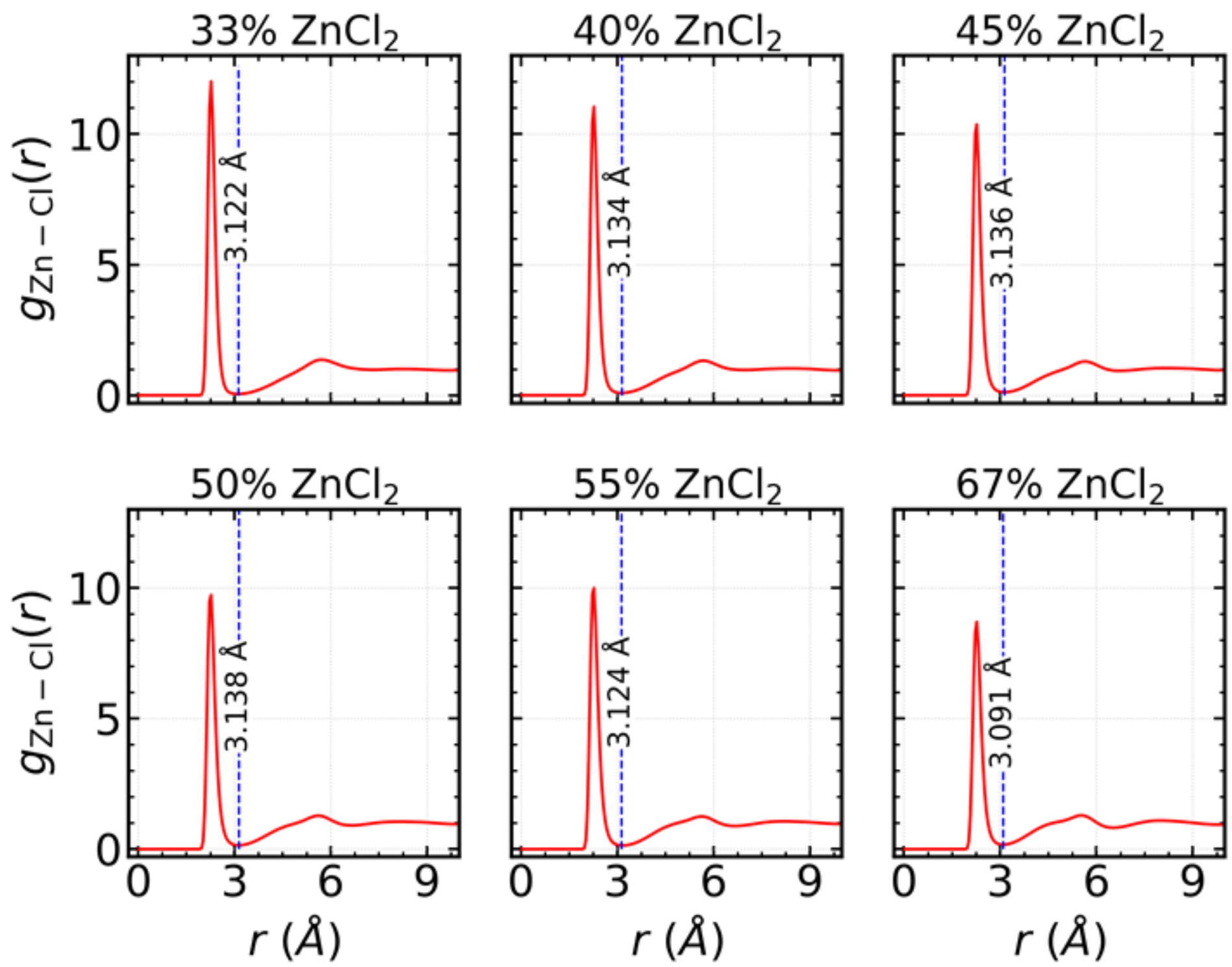

Figure S1. Radial distribution functions (RDF) for the Zn-Cl pair in $ZnCl_2$-KCl. The first minimum of the RDFs for each composition is shown with a blue vertical line.

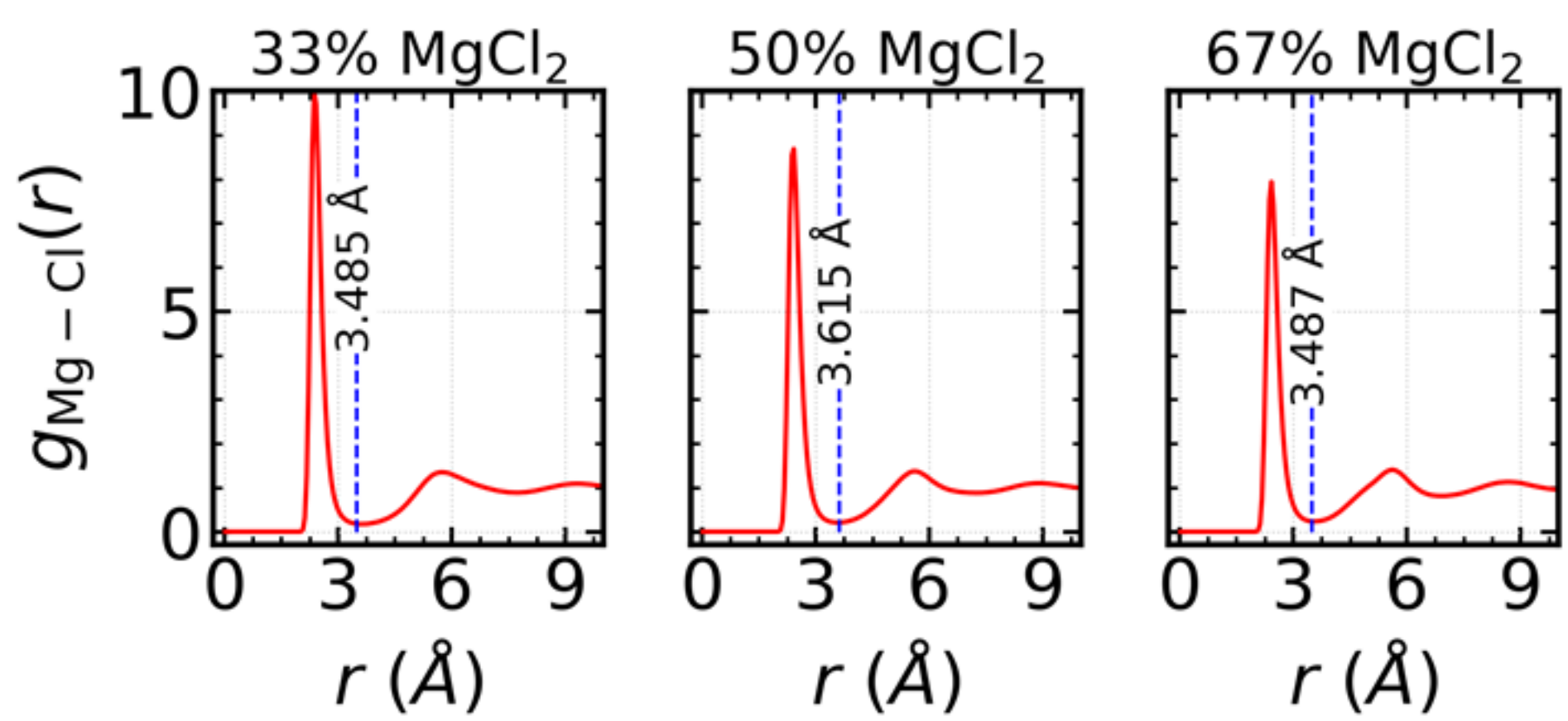

Figure S2. Radial distribution functions (RDF) for the Mg-Cl pair in $MgCl_2$-KCl. The first minimum of the RDFs for each composition is shown with a blue vertical line.

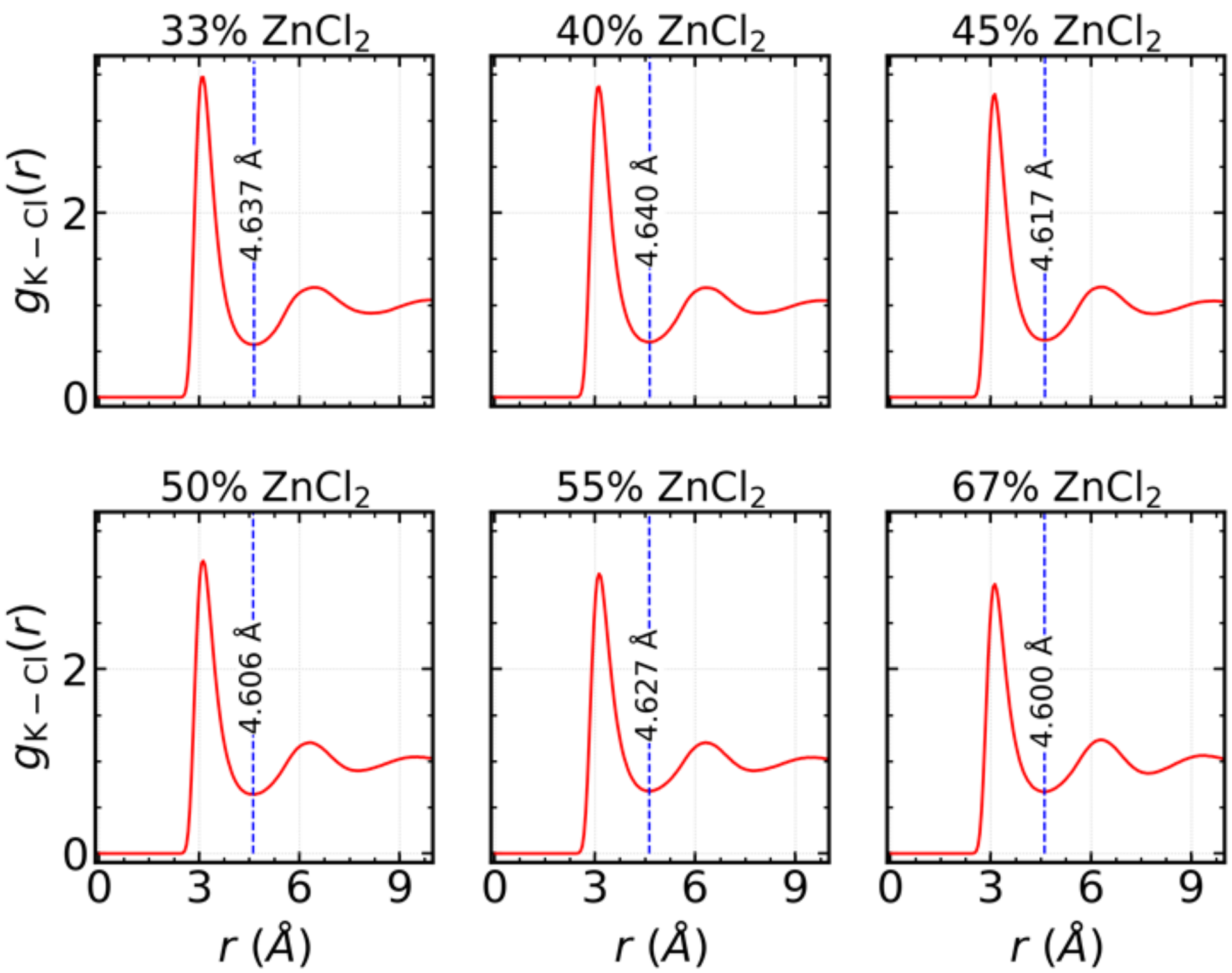

Figure S3. Radial distribution functions (RDF) for the K-Cl pair in $ZnCl_2$-KCl. The first minimum of the RDFs for each composition is shown with a blue vertical line.

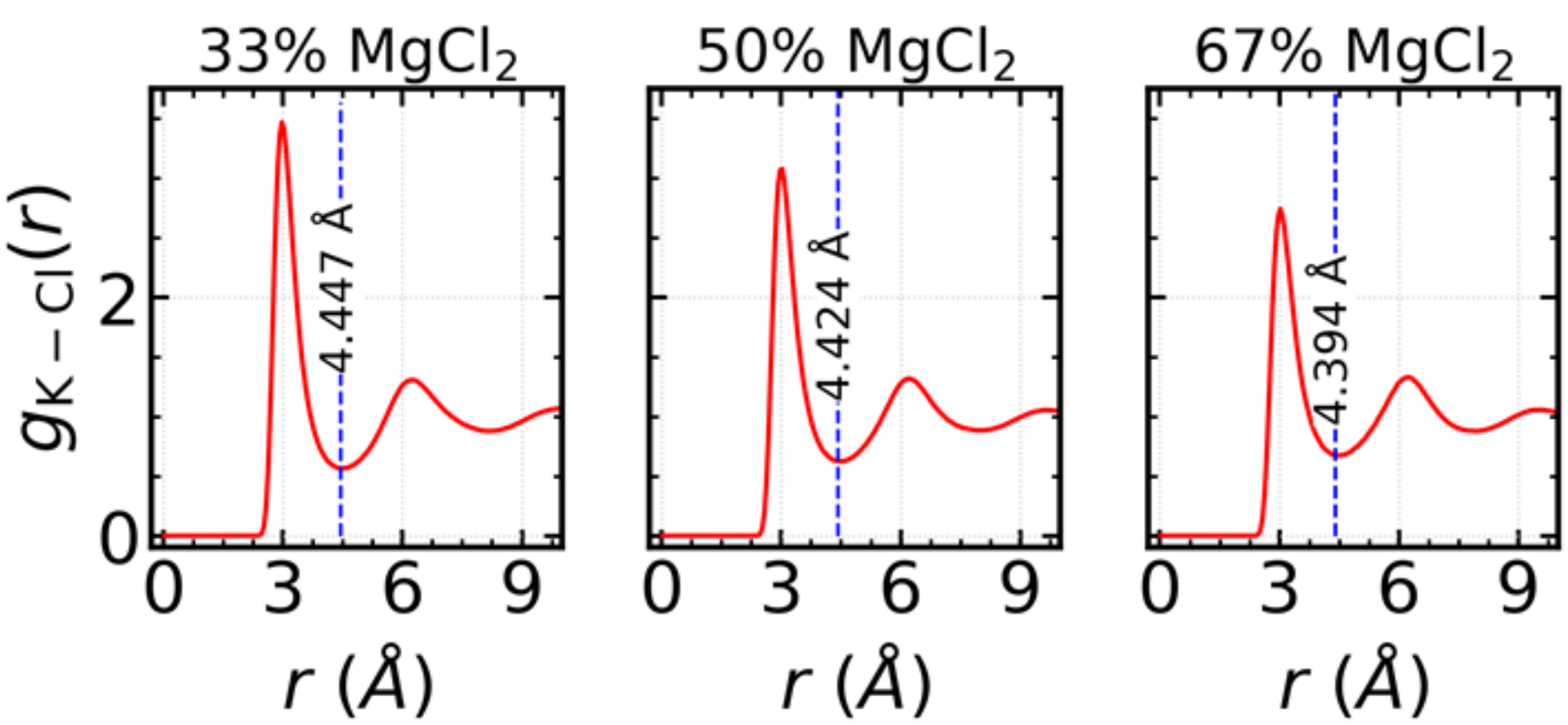

Figure S4. Radial distribution functions (RDF) for the K-Cl pair in $MgCl_2$-KCl. The first minimum of the RDFs for each composition is shown with a blue vertical line.

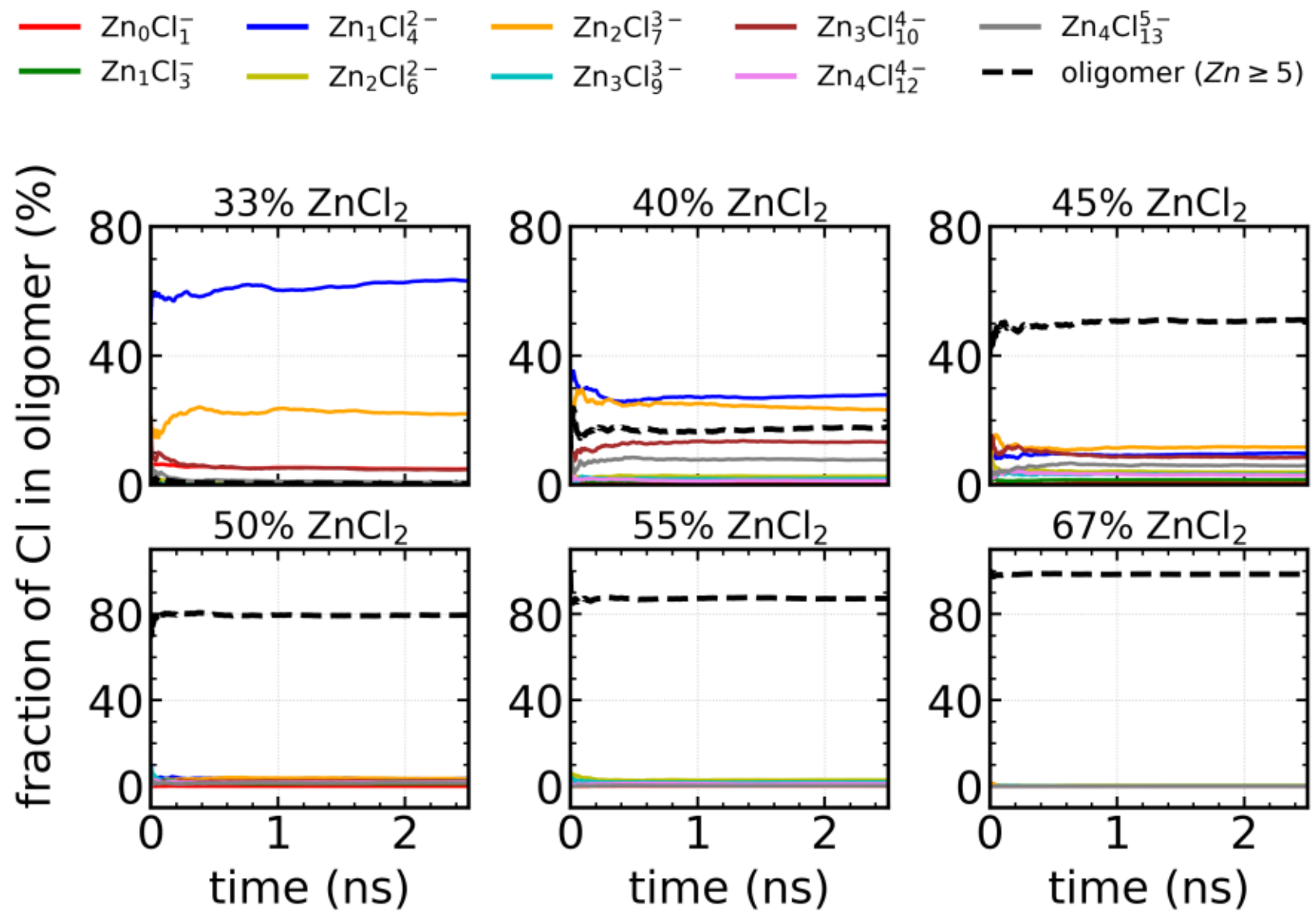

Figure S5. Running averages of the fraction of each species obtained from cluster analysis of MD trajectories of $ZnCl_2$–KCl mixtures. Each curve reports the percentage of Cl^- ions coordinated within a given $Zn_nCl_m^{(m-2n)-}$ motif or within larger oligomeric clusters (Zn ≥ 5).

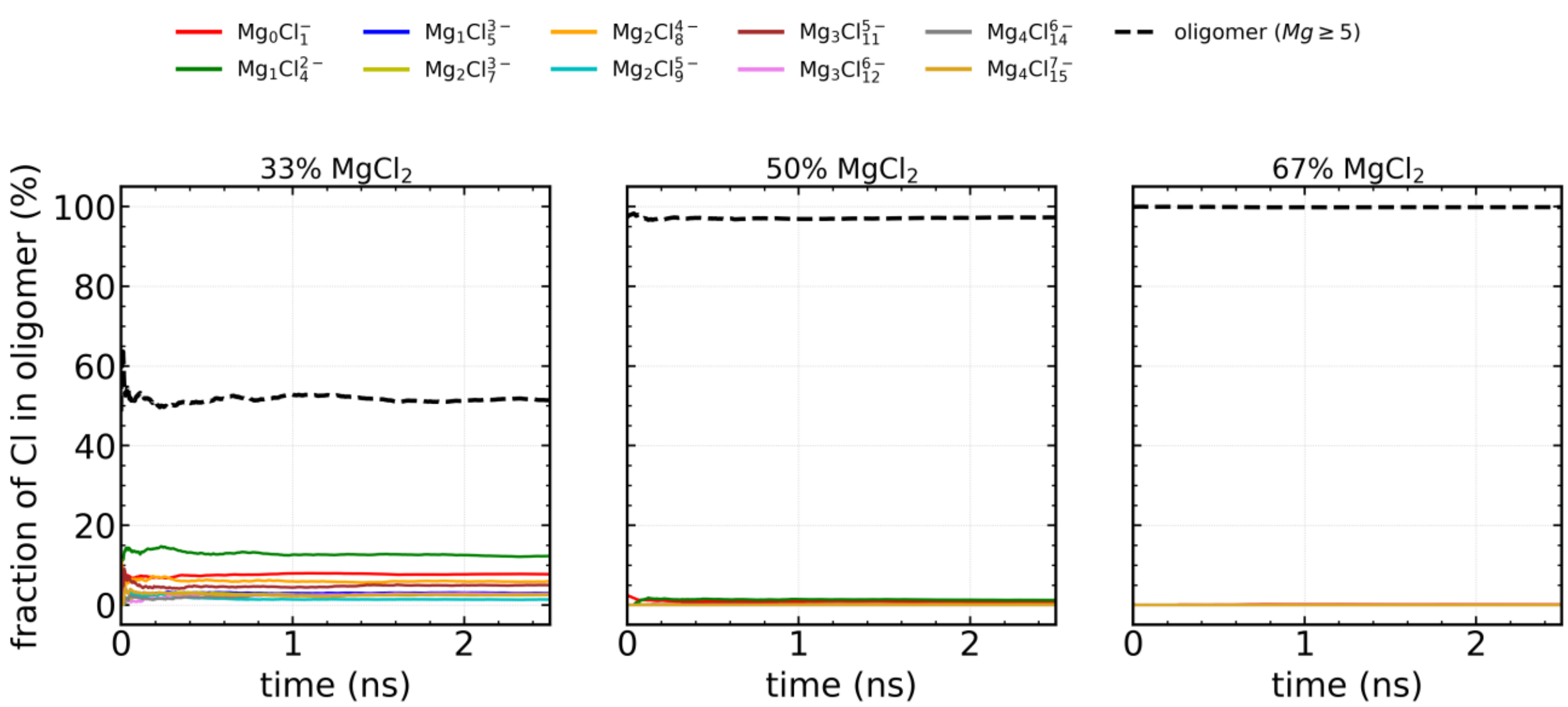

Figure S6. Running averages of the fraction of each species obtained from cluster analysis of MD trajectories of MgCl-KCl mixtures. Each curve reports the percentage of Cl^- ion coordinated within a given $Mg_nCl_m^{(m-2n)}$ motif or within larger oligomeric clusters (Mg ≥ 5).

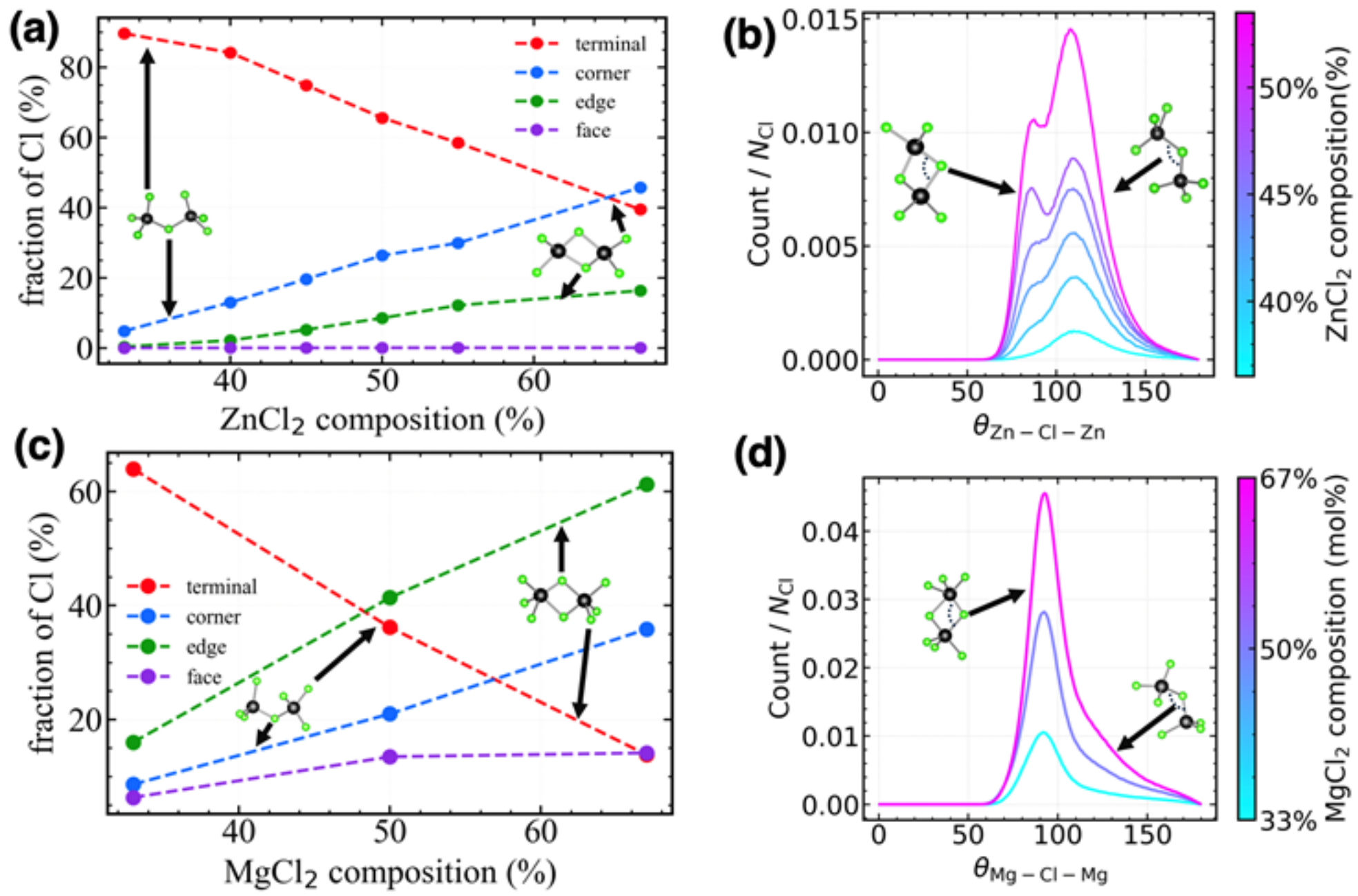

Figure S7. Population of terminal, corner-, edge-, and face-sharing chlorides as a function of $ZnCl_2$ (a) and $MgCl_2$ (c) concentration. (b) Zn-Cl-Zn and (d) Mg-Cl-Mg angular distribution functions normalized by the number of shared Cl-.

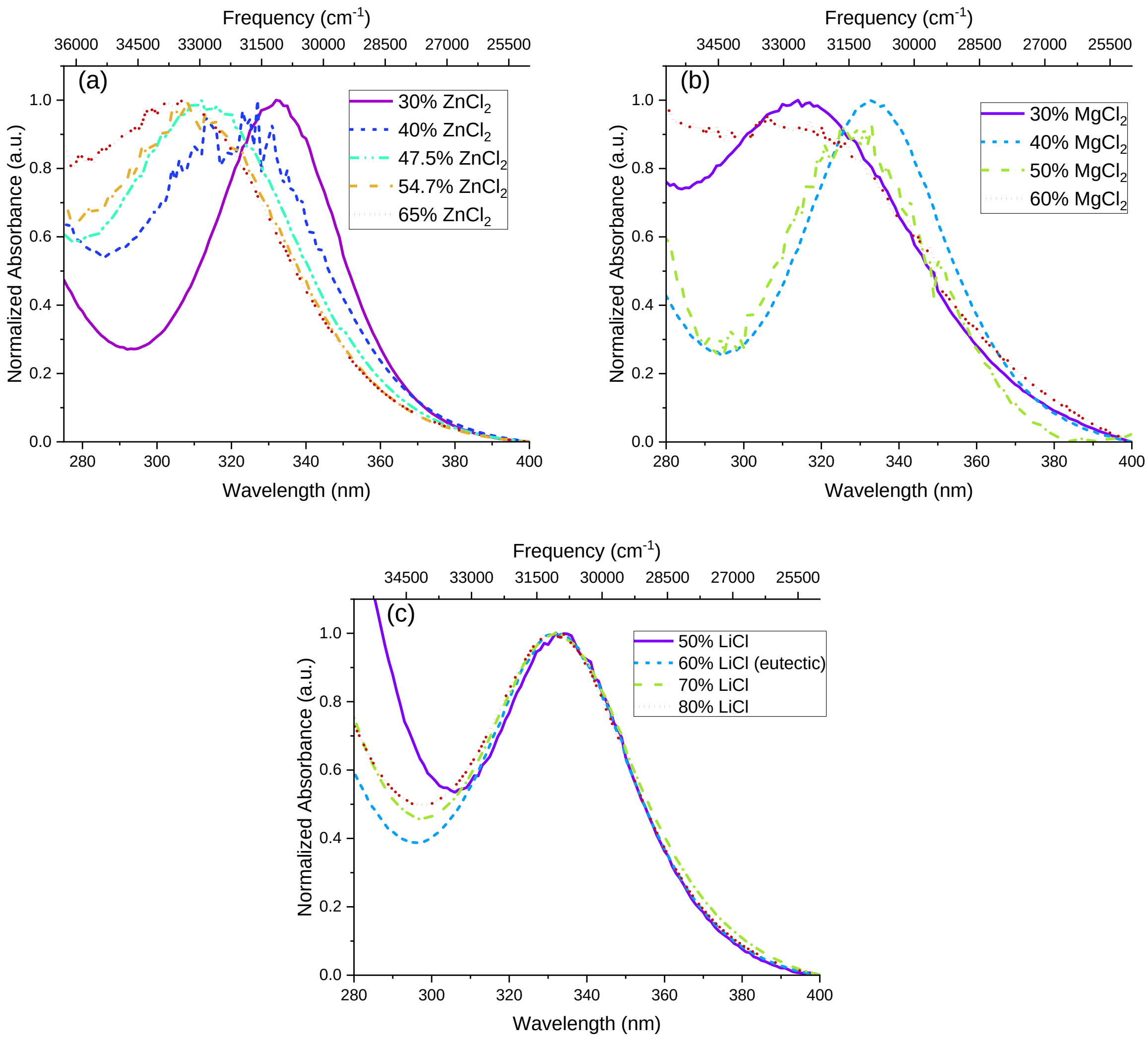

Figure S8. Normalized, blank subtracted Bi^{3+} peak in (a) select $ZnCl_2$-KCl salts at 450 °C, (b) $MgCl_2$-KCl salts at 550 °C, and (c) LiCl-KCl salts at 550 °C. $ZnCl_2$-KCl compositions >65 mol% $ZnCl_2$ were omitted from this figure for clarity.

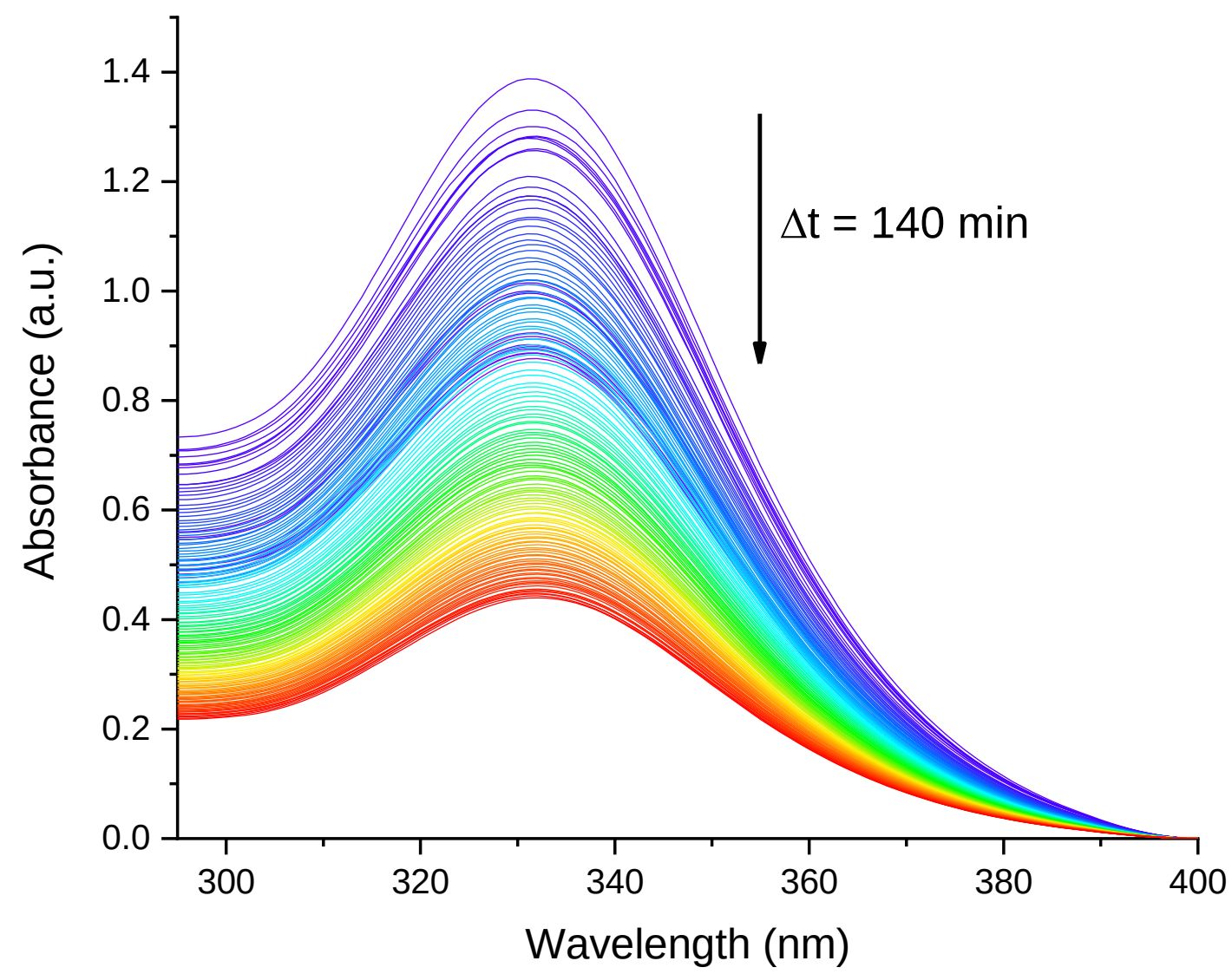

Figure S9. Spectral evolution of Bi^{3+} peak in eutectic LiCl-KCl during heating for 140 minutes at 550 °C.

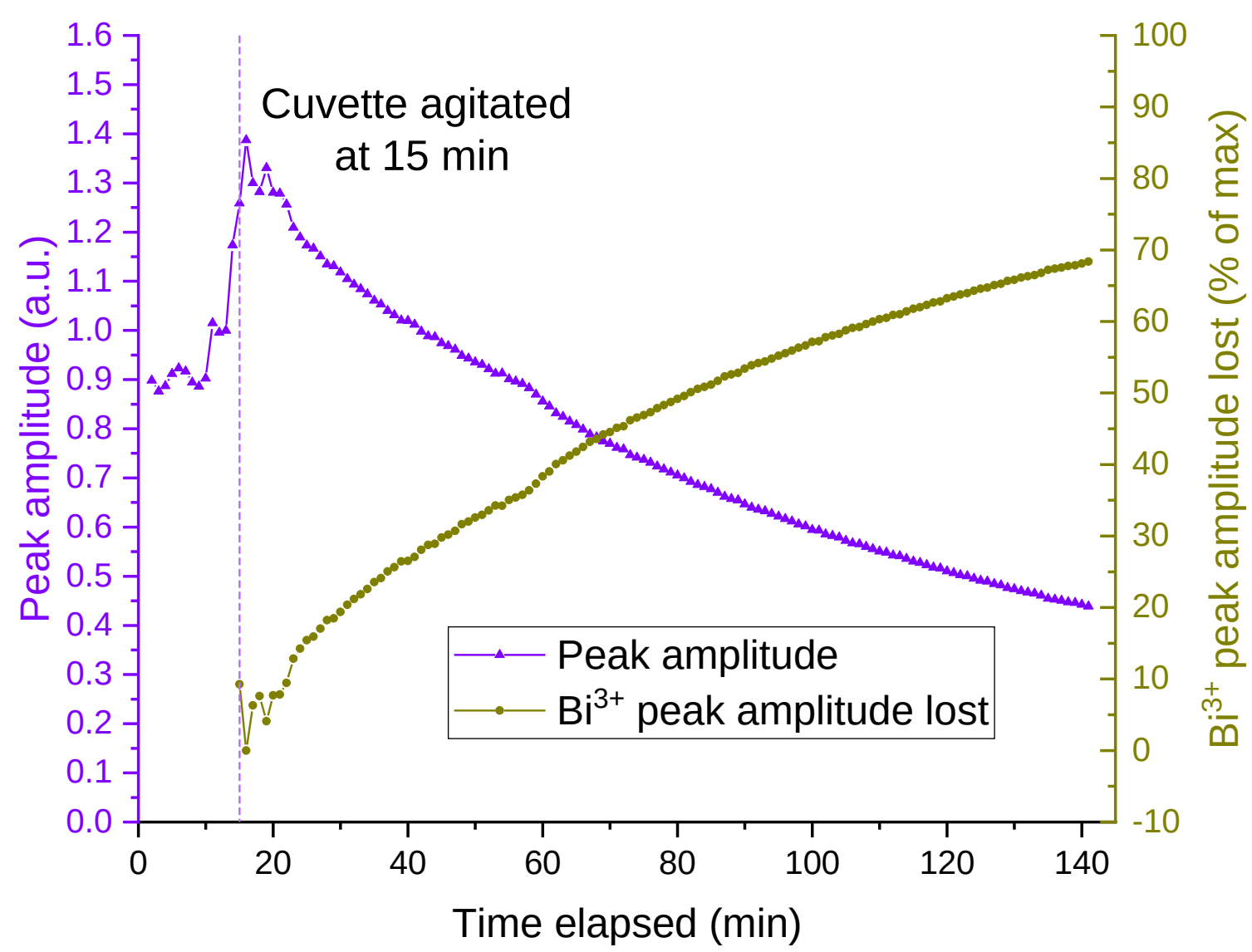

Figure S10. Bi^{3+} peak amplitude in eutectic LiCl-KCl at 550 °C plotted as a function of elapsed time since the addition of $BiCl_3$. Initial inhomogeneity is evident by the instability of peak amplitude for the first 15 minutes. The cuvette was shaken to fully mix the salt after 15 minutes. The relative amount of Bi^{3+} lost, in units of percent relative to the maximum measured amplitude, is plotted as well.

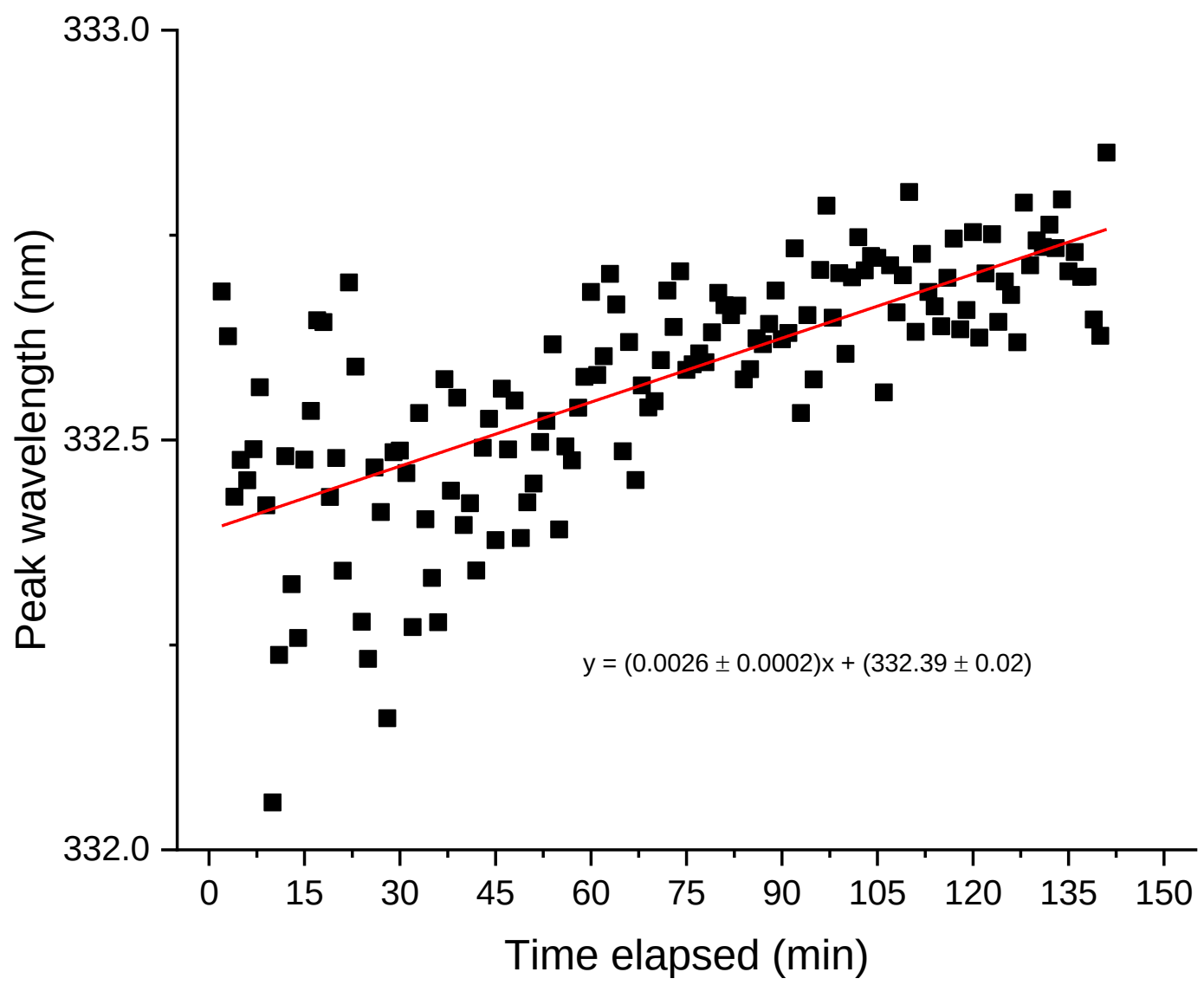

Figure S11. Evolution in Bi^{3+} peak wavelength in eutectic LiCl-KCl during heating for 140 minutes at 550 °C. Measurements remained within one wavelength of variation during the course of measurement.

Table S1. Bismuth $^1S_0 \rightarrow {}^3P_1$ peak frequencies for each salt system studied

	Peak frequency (cm^{-1})												
	$ZnCl_2$-KCl			$MgCl_2$-KCl					LiCl-KCl				
**% MCl_n (100-%KCl)**	350 °C	400 °C	450 °C	500 °C	525 °C	550 °C	575 °C	585 °C	450 °C	475 °C	500 °C	525 °C	550 °C
30			30102	30006	29969	29891							
40		31190	30957	30370	30246	30246	30285	30209					
47.5	32230	32011	31816										
50				30932	31008	30813	30809	30746	30081	30043		30028	29947
54.7	32389	32251	32045										
60			31919	31274	31293	31045			30132	30083	30072	30044	30070
65	32569	32355	32176				30957	31023					
70			32003					31064		30075		30009	30084
75			32188										
80			32130						30147	30163		30098	30076
